# Sex Differences in Carotid Atherosclerosis: A Systematic Review and Meta-Analysis

**DOI:** 10.1161/STROKEAHA.122.041046

**Published:** 2022-11-29

**Authors:** Dianne H.K. van Dam-Nolen, Nina C.M. van Egmond, Peter J. Koudstaal, Aad van der Lugt, Daniel Bos

**Affiliations:** 1 Department of Radiology and Nuclear Medicine (D.H.K.v.D.-N., N.C.M.v.E., A.v.d.L., D.B.), Erasmus University Medical Center Rotterdam, the Netherlands.; 2 Department of Neurology (D.H.K.v.D.-N., P.J.K.), Erasmus University Medical Center Rotterdam, the Netherlands.; 3 the Department of Epidemiology (D.B.), Erasmus University Medical Center Rotterdam, the Netherlands.

**Keywords:** atherosclerotic plaque, atherosclerosis, carotid stenosis, computed tomography angiography, ischemic stroke, magnetic resonance imaging, sex characteristics

## Abstract

Over the last decades, several individual studies on sex differences in carotid atherosclerosis have been performed covering a wide range of plaque characteristics and including different populations. This systematic review and meta-analysis aims to summarize previously reported results on sex differences in carotid atherosclerosis and present a roadmap explaining next steps needed for implementing this knowledge in clinical practice.

**Methods:**

We systematically searched PubMed, Embase, Web of Science, Cochrane Central, and Google Scholar for eligible studies including both male and female participants reporting prevalence of imaging characteristics of carotid atherosclerosis and meta-analyzed these studies. Studies had to report at least the following: (1) calcifications; (2) lipid-rich necrotic core; (3) intraplaque hemorrhage; (4) thin-or-ruptured fibrous cap; (5) plaque ulceration; (6) degree of stenosis; (7) plaque size; or (8) plaque inflammation. We prespecified which imaging modalities had to be used per plaque characteristic and excluded ultrasonography.

**Results:**

We included 42 articles in our meta-analyses (ranging from 2 through 23 articles per plaque characteristic). Men had more frequently a larger plaque compared to women and, moreover, had more often plaques with calcifications (odds ratio=1.57 [95% CI, 1.23–2.02]), lipid-rich necrotic core (odds ratio=1.87 [95% CI, 1.36–2.57]), and intraplaque hemorrhage (odds ratio=2.52 [95% CI, 1.74–3.66]), or an ulcerated plaque (1.81 [95% CI, 1.30–2.51]). Furthermore, we found more pronounced sex differences for lipid-rich necrotic core in symptomatic opposed to asymptomatic participants.

**Conclusions:**

In this systematic review and meta-analysis, we demonstrate convincing evidence for sex differences in carotid atherosclerosis. All kinds of plaque features—plaque size, composition, and morphology—were more common or larger in men compared to women. Our results highlight that sex is an important variable to include in both study design and clinical-decision making. Further investigation of sex-specific stroke risks with regard to plaque composition is warranted.

Carotid atherosclerosis is considered the underlying cause in 10% to 15% of all ischemic strokes worldwide.^1^ An important aspect with regard to the occurrence of ischemic strokes is that men have higher lifetime risks for ischemic stroke and have more often strokes related to large-artery atherosclerosis, while cardioembolic strokes are more common among women.^2^ Furthermore, trials on carotid endarterectomy reported that perioperative stroke and death risks are higher among women than among men and moreover that women benefit less from surgery.^3^ Sex differences in severity and composition of carotid atherosclerosis could explain differences in stroke incidence, treatment benefit, and complication rate between men and women.

Over the last decades, several studies on sex differences in carotid atherosclerosis have been performed, covering a wide range of patients (asymptomatic versus symptomatic patients and mild versus severe atherosclerosis) and assessments of atherosclerotic disease (plaque size, degree of stenosis, or plaque composition). Moreover, those individual studies used different levels of adjustments for confounders. The aim of this study is to systematically review all literature on sex differences in carotid atherosclerosis in order to provide a comprehensive overview of sex differences in carotid plaque composition and morphology. Additionally, we aim to meta-analyze previously reported results and to present a roadmap explaining next steps that are needed for implementing this knowledge in clinical practice.

## Methods

This systematic review and meta-analysis was performed according to the PRISMA (Preferred Reporting Items for Systematic Reviews and Meta-Analysis) guidelines.^4^ Data not published within the article are available from the corresponding author upon reasonable request.

### Search Strategy

The search query was designed with an expert librarian at the medical library of the Erasmus University Medical Center to capture all citations reported from inception to February, 2022. To identify eligible studies within PubMed, Embase, Web of Science, Cochrane Central, and Google Scholar, the following keywords were used in combination with the Boolean operators OR and AND: atherosclerotic plaque, atherosclerosis, calcification, calcinosis, vascular calcification, stenosis and carotid artery, carotid artery diseases, carotid stenosis, and sex characteristics, sex distribution, sex factors, sex, gender, men, man, male, women, woman, female, and radiography, tomography, mri, cta, ct, pet, angiography. The full search queries are listed in the Supplemental Material.

### Study Selection

Studies were included if they enrolled both male and female participants, reported prevalence of imaging characteristics of carotid plaque composition, morphology, and size, and were written in English language. After removing duplicate reports, 2 independent reviewers (D.v.D.N. and N.v.E.) screened the studies by title and abstract. Conflicts were resolved by consensus between the 2 reviewers. Studies had to report at least one of the following plaque characteristics: (1) calcifications; (2) lipid-rich necrotic core (LRNC); (3) intraplaque hemorrhage (IPH); (4) thin-or-ruptured fibrous cap; (5) plaque ulceration; (6) degree of stenosis; (7) plaque size (ie, plaque thickness, area, or volume); and (8) plaque inflammation. We excluded studies that only investigated prevalence of carotid atherosclerosis and did not examine plaque composition or morphology. We prespecified which imaging modalities had to be used as minimum requirement per plaque characteristic (Table S1) and excluded those articles that used other techniques.^5,6^ For instance, we decided to exclude ultrasonography as modality because of high interrater variability, the ability of more reliable modalities like computed tomography or magnetic resonance imaging, and to end up with a limited and feasible overview of articles. We also excluded studies that investigated the prevalence of calcifications on dental panoramic tomographies, since dental panoramic tomography is not the gold standard. Furthermore, we excluded studies solely reporting measures of effect size without raw prevalences. Lastly, letters and congress abstracts were excluded.

Since we included sex as a criterion in our search term and to overcome that we therefore missed relevant articles that do not explicitly mention this key word in title or abstract, we also hand-searched all references from preceding included articles to screen for additional relevant studies.

### Assessment of Risk of Bias

We assessed the quality of included studies using an adapted version of the Newcastle-Ottawa Scale.^7^ For this study, we developed a customized version of the scale including criteria with regard to the used cut-off for carotid atherosclerosis, separate versus simultaneous analyses of both carotid arteries, stratification for symptomatic versus asymptomatic patients, and the assessment of plaque characteristics (see Supplemental Methods). For the domains (1) selection, (2) information, and (3) outcome, all studies were classified as low, possible, or high risk for bias. If studies had a high risk of bias in ≥2 domains, they were excluded for meta-analyses.

### Statistical Analyses

The main outcomes of interest were prevalence and volume of aforementioned plaque characteristics, all stratified for sex. These data were collected from the included studies and presented as odds ratio (OR) or β with 95% CI. The effect estimates were pooled using random-effect meta-analyses. Heterogeneity between studies was assessed using the I^2^ index. We additionally stratified the analyses for asymptomatic versus symptomatic (ie, recent history of stroke or transient ischemic attack) arteries, based on the information provided in the articles. Statistical analyses were conducted in R statistical software (version 4.1.2; R Foundation for Statistical Computing, Vienna, Austria) using the package “meta.”

## Results

**Figure 1.**
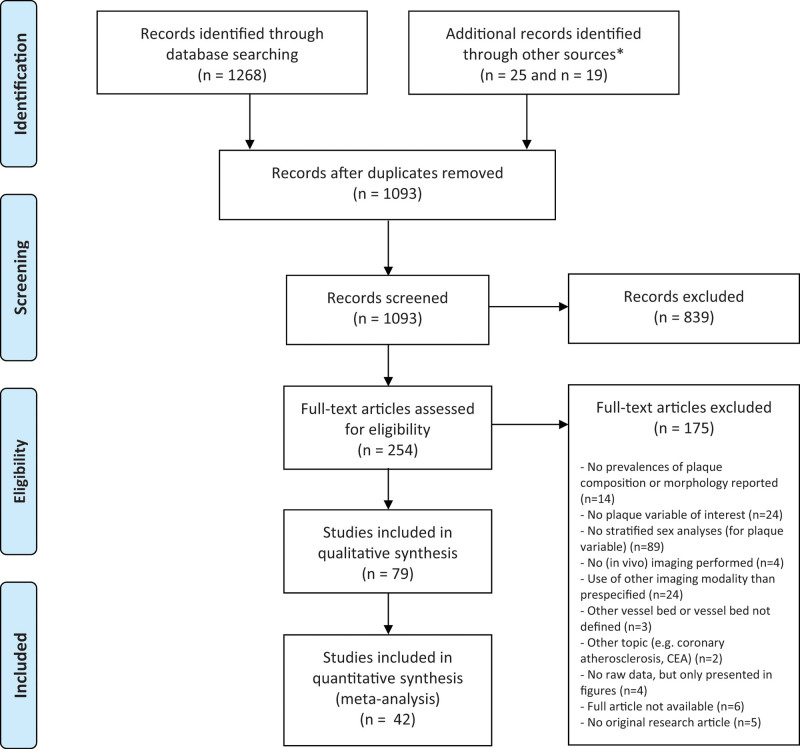
**Flowchart of identification and selection of included articles.** *We identified additional records by screening the reference lists with a specific attention for plaque ulceration (n=25) and with attention for other relevant articles (n=19).

We identified 530 citations from Embase, 359 from PubMed, 197 from Web of Science, 82 from Cochrane Central, and 100 from Google Scholar. Removal of duplicate reports resulted in 1074 unique citations that were screened by title and abstract. From these, 235 were read in full resulting in a final selection of 60 articles.^8–67^ Main reasons for exclusion were absence of reporting stratified sex analyses regarding the plaque characteristics (n=89), use of another imaging modality than prespecified (n=24), and no inclusion of a plaque variable of interest (n=24), see Figure 1. Table S2 presents the details of the final included articles. For the meta-analyses per plaque characteristic, we excluded 24 articles, because of overlap with other studies performed in the same study cohort, selecting the one that included most participants. After hand-searching reference lists of the included articles in the meta-analyses for other relevant articles, we included an additional 19 articles^68–86^ of which 10 were eligible for meta-analyses. Table S3 reports the quality assessment scores per included study. No study had a high risk of bias in ≥2 domains and therefore all these studies could be included in the meta-analyses.

### Plaque Size

**Figure 2.**
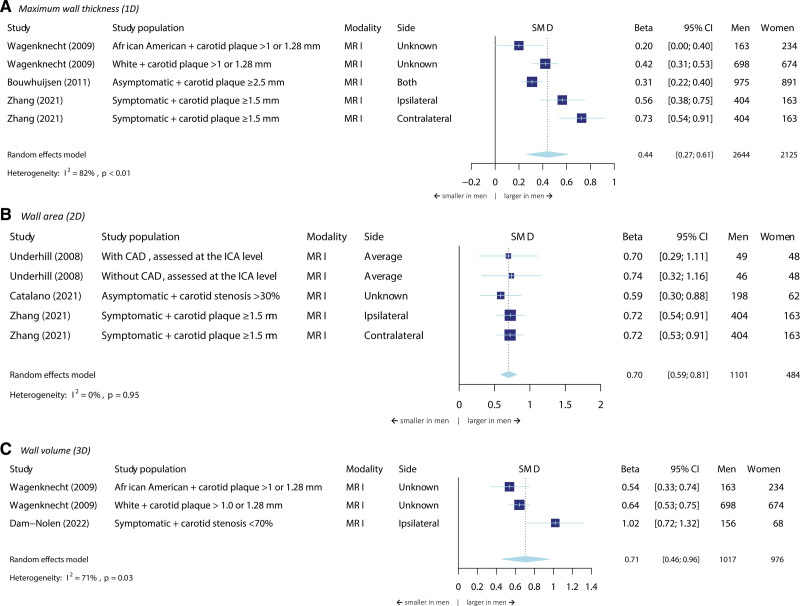
**Meta-analysis on the association between sex and plaque size.** The size of the box, which represents the beta, is proportional to the weight of the study. The diamond is the result of the random-effect meta-analysis. Wagenknecht et al (2009) included both symptomatic and asymptomatic participants. CAD indicates coronary artery disease; ICA, internal carotid artery; MRI, magnetic resonance imaging; and SMD, standardized mean difference.

Six different studies were included in the meta-analyses on the relation between sex and plaque size. Figure 2 shows the results per measure of plaque size, that is, maximum wall thickness as 1-dimensional (1D) size, wall area as 2D size, and wall volume as 3D size. In the meta-analyses, all 3 characteristics were more likely to be larger in men opposed to women (β=0.44 [95% CI, 0.27–0.61] for maximum wall thickness; β=0.70 [95% CI, 0.59–0.81] for wall area; and β=0.71 [95% CI, 0.46–0.96] for wall volume). Conversely, the normalized wall index, which accounts for the total vessel size, did not statistically significant differ between male and female participants (Figure S1). However, the heterogeneity of the studies in this meta-analysis was high (I^2^=88%, *P* value<0.01). For instance, the study that showed a statistically significant result in both ipsilateral and contralateral arteries was done in patients that had a recent stroke or transient ischemic attack.

With regard to the degree of stenosis, we could meta-analyze the stenosis severity within 2 subgroups: with a stenosis of either 50% to 69% or 70% to 99% (Figure S2). There was high heterogeneity in analyzed variables among studies, for instance due to different cut-off values. Another important reason why merely 3 studies could be included in the meta-analyses was the type of imaging modality (often ultrasonography). We found no statistically significant sex difference for stenosis of 50% to 69%. However, high-grade stenosis of 70% to 99% was more often seen in men than in women (OR=1.69 [95% CI, 1.30–2.21]).

We were unable to meta-analyze plaque size for asymptomatic versus symptomatic participants because we lacked an adequate number studies that stratified for these 2 groups.

### Plaque Composition

**Figure 3.**
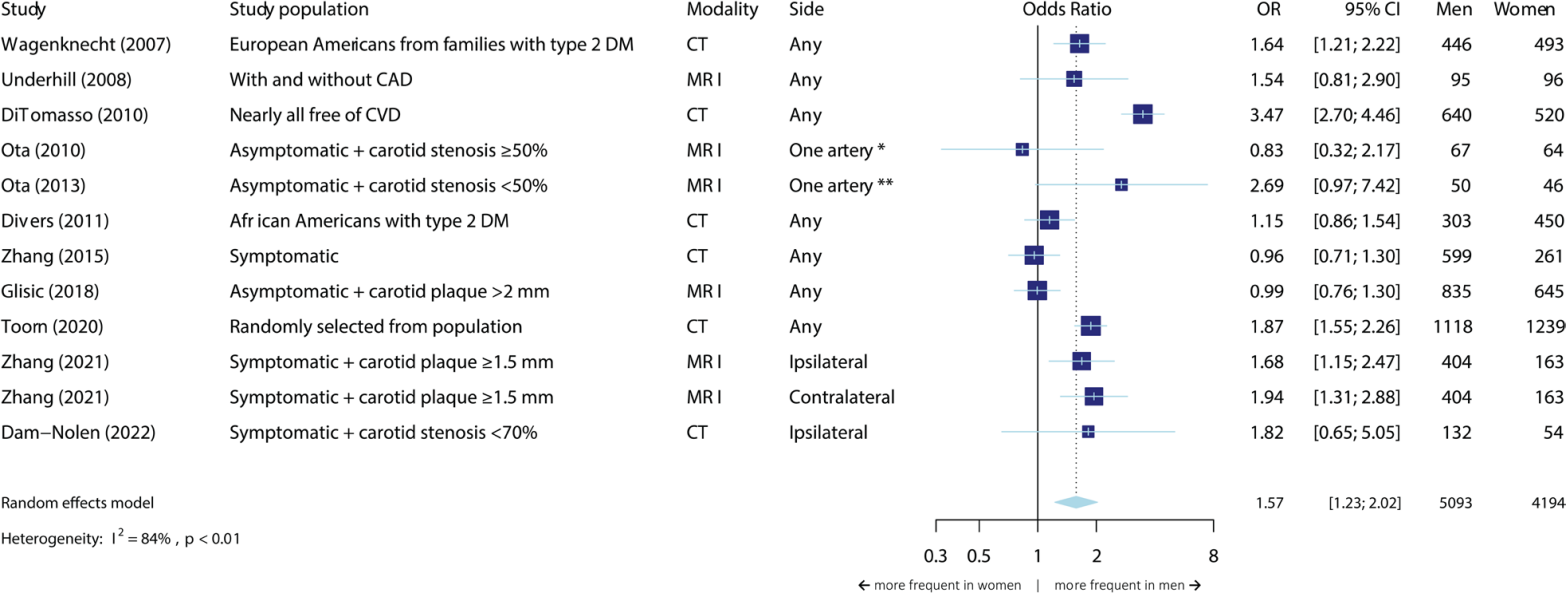
**Meta-analysis on the association between sex and the presence of carotid calcifications.** The size of the box, which represents the odds ratio, is proportional to the weight of the study. The diamond is the result of the random-effect meta-analysis. CAD indicates coronary artery disease; CT, computed tomography; CVD, cardiovascular disease; DM, diabetes; MRI, magnetic resonance imaging; and OR‚ odds ratio. *Most severely stenotic side. **Side with stenosis <50%.

Figures 3 through 5 show the meta-analyses for sex differences in plaque composition, specifically for the presence of calcifications, LRNC, and IPH. For all components, we found a higher prevalence in men than in women.

**Figure 4.**
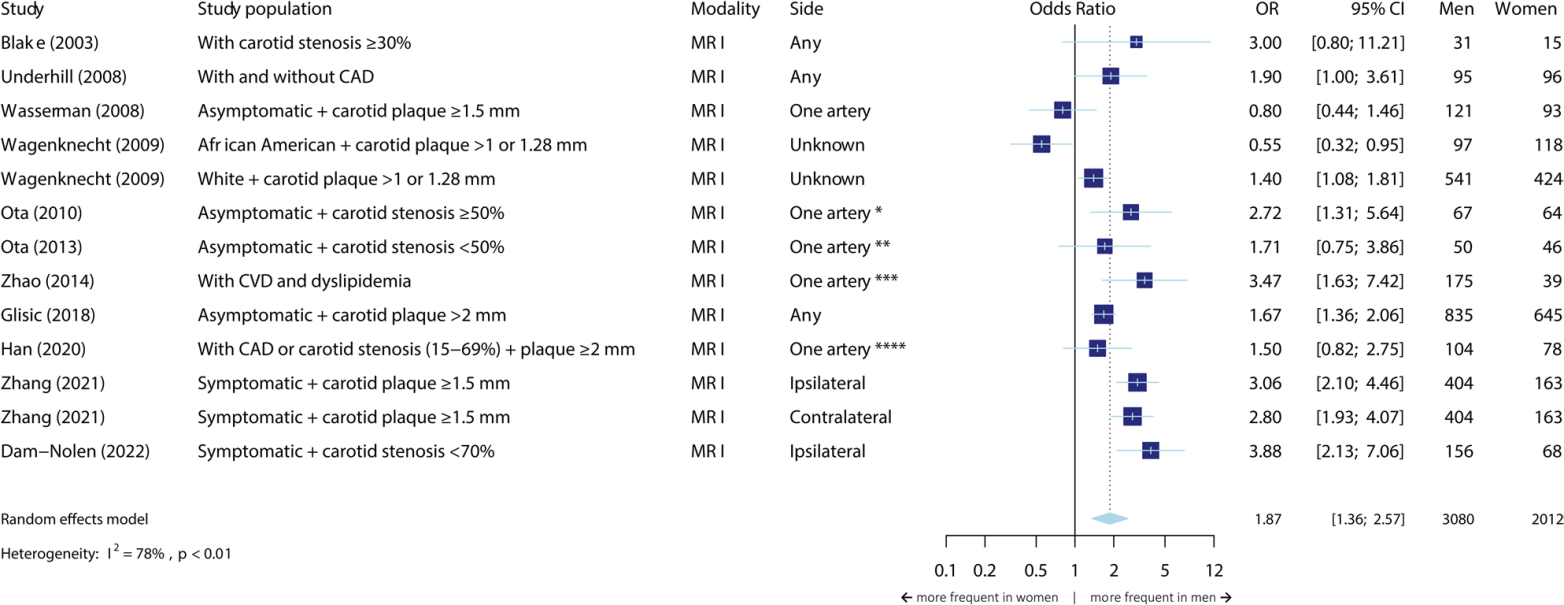
**Meta-analysis on the association between sex and the presence of lipid-rich necrotic core.** The size of the box, which represents the odds ratio, is proportional to the weight of the study. The diamond is the result of the random-effect meta-analysis. CAD indicates coronary artery disease; CVD, cardiovascular disease; MRI, magnetic resonance imaging; and OR‚ odds ratio. *Most severely stenotic side. **Side with stenosis <50%. ***Side with largest plaque. ****Side with greatest thickness.

**Figure 5.**
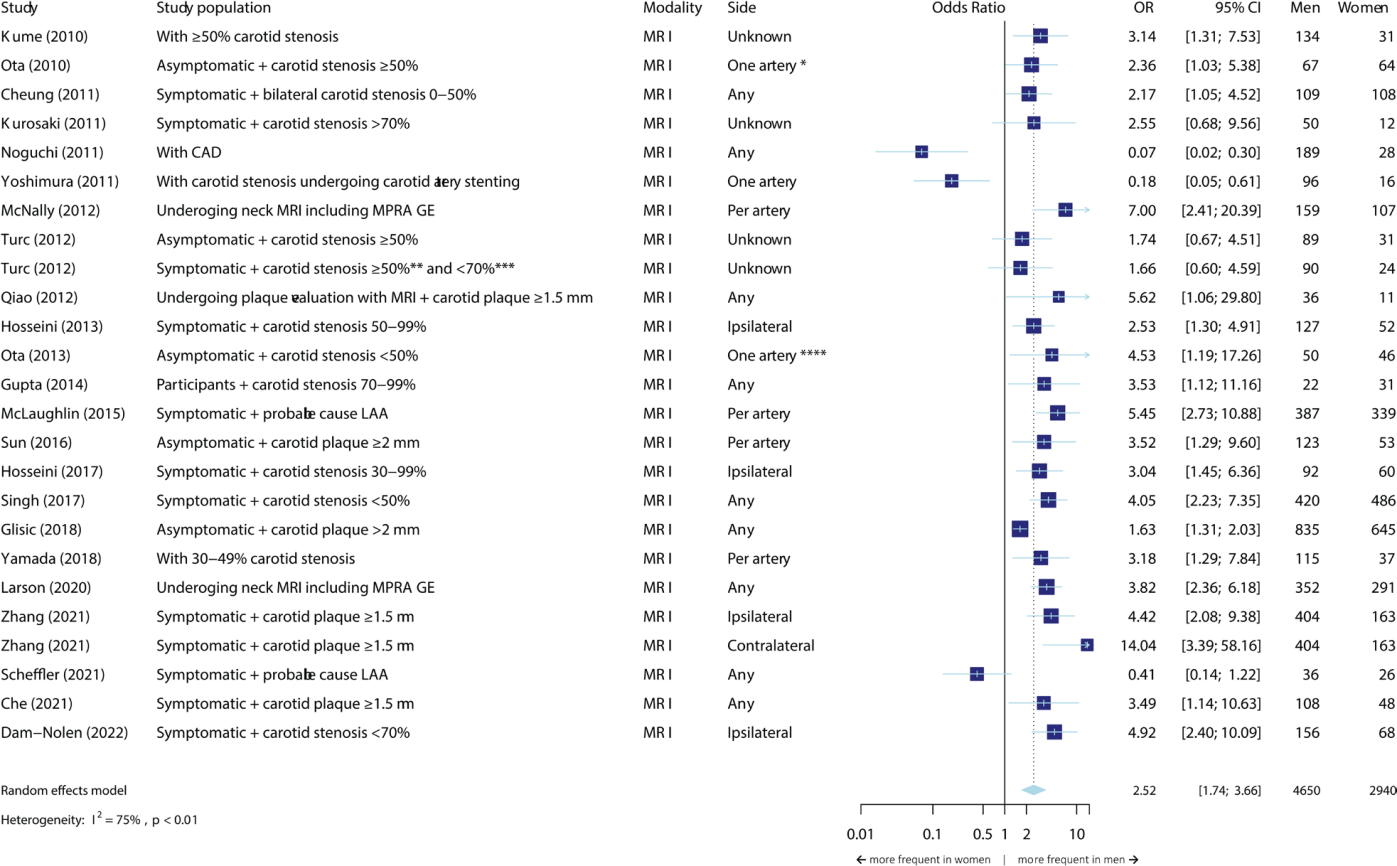
**Meta-analysis on the association between sex and the presence of intraplaque hemorrhage.** The size of the box, which represents the odds ratio, is proportional to the weight of the study. The diamond is the result of the random-effect meta-analysis. CAD indicates coronary artery disease; CVD, cardiovascular disease; MRI, magnetic resonance imaging; and OR, odds ratio. *Most severely stenotic side. **According to the ECST criteria. ***According to the NASCET criteria. ****Side with stenosis <50%.

#### Calcifications

Eleven studies were included in the meta-analyses for the presence of carotid calcifications (Figure 3). The total number of participants was 9287 and included both symptomatic and asymptomatic arteries. We found a statistically significant difference between men and women for having carotid calcifications. There was substantial heterogeneity between studies (I^2^=84%), which may be explained by differences in studies with regard to selection based on prevalent carotid atherosclerosis. When we stratified for this criterion, we found an OR of 1.63 (95% CI, 1.12–2.38) for having carotid calcifications among the studies without selection on prevalent atherosclerosis^17,47,51,59,65,74^; and an OR of 1.47 (95% CI, 1.06–2.05) for studies with selection on prevalent atherosclerosis.^21,34,35,49,66^ Regarding the amount of calcifications, we found that men have higher calcification volumes than women (β=0.37 [95% CI, 0.25–0.48]; Figure S3). However, we found no sex difference in calcification percentage, that is, the amount of calcification relative to the total plaque volume (β=0.01 [95% CI, −0.15 to 0.16]).

#### Lipid-Rich Necrotic Core

Eleven studies were included in the meta-analyses for the presence of LRNC (Figure 4) with a total number of participants of 5092. Most studies used presence of carotid atherosclerosis as an inclusion criterion (10 out of 11). Based on the meta-analyses, men are more likely to have a LRNC (OR=1.87 [95% CI, 1.36–2.57]). With regard to the amount of lipid, we found no statistically significant sex difference for absolute lipid volumes (β=0.05 [95% CI, −0.17 to 0.26]; Figure S4). In contrast, regarding relative lipid volumes, men do more often have higher volumes (β=0.42 [95% CI, 0.31 to 0.53]).

#### Intraplaque Hemorrhage

Twenty-three studies were included in the meta-analyses for the presence of IPH (Figure 5) comprising 7590 participants in total. Ten studies included only symptomatic patients and 5 studies only asymptomatic participants. All studies except 6 reported that IPH is more common in men than in women. When we pooled the effect estimates of the included studies, we found an OR of 2.52 (95% CI, 1.74–3.66) for IPH presence in men compared to women (Figure 5). Regarding the amount of IPH, both absolute and relative volumes of IPH were significantly higher in men compared to women (β=0.26 [95% CI, 0.01–0.52], β=0.26 [95% CI, 0.13–0.38], respectively; Figure S5).

When we stratified the meta-analyses based on the inclusion of asymptomatic versus symptomatic participants (Figures S6 through S8), we found more pronounced sex differences in the symptomatic compared to the asymptomatic group for LRNC (OR=3.27 [95% CI, 2.38–4.50] versus OR=1.79 [95% CI, 1.16–2.76]; *P* value for subgroup differences=0.03).

### Plaque Morphology

**Figure 6.**
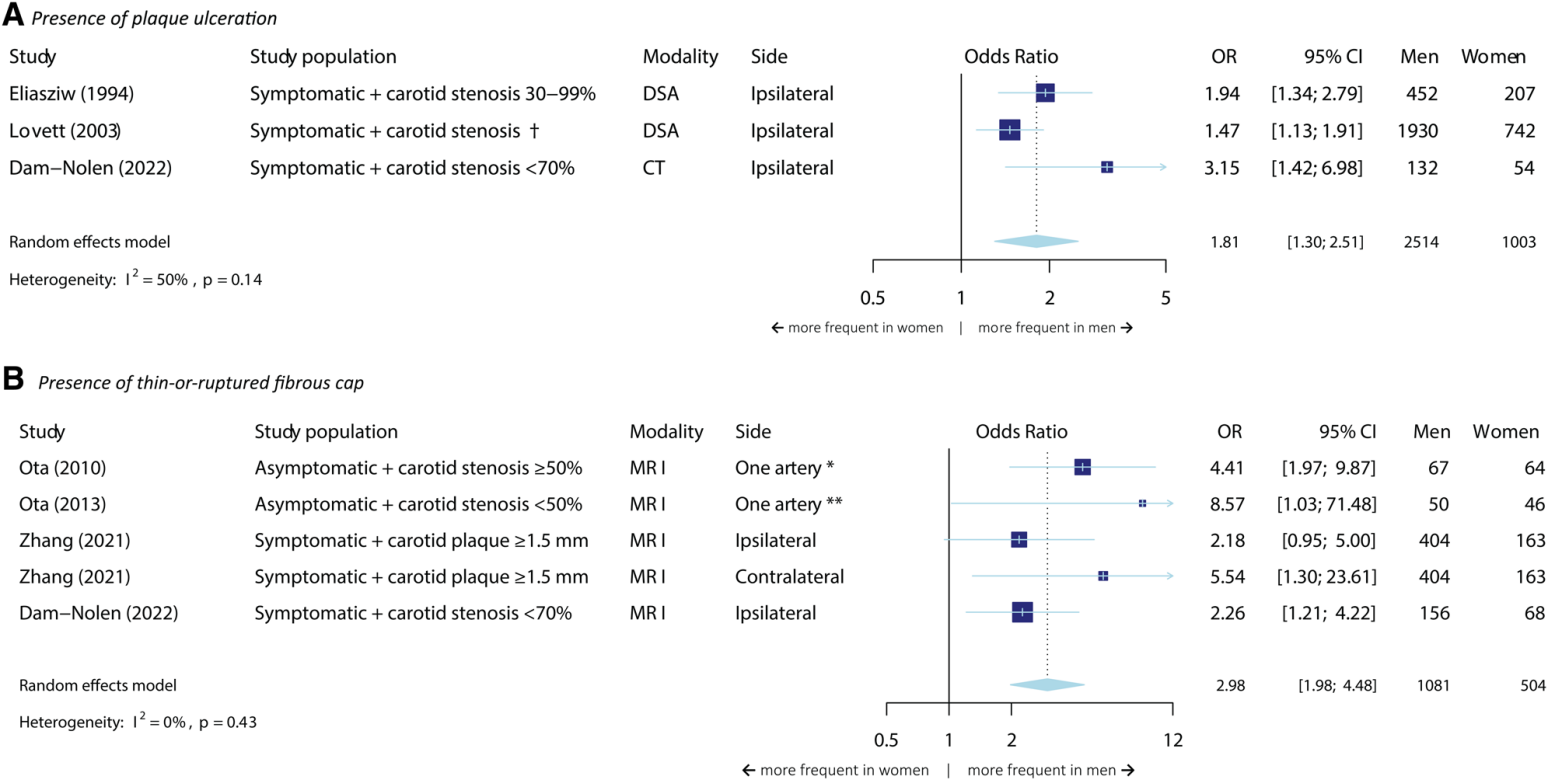
**Meta-analysis on the association between sex and the presence of plaque ulceration and a thin-or ruptured fibrous cap.** The size of the box, which represents the odds ratio, is proportional to the weight of the study. The diamond is the result of the random-effect meta-analysis. CT indicates computed tomography; DSA, digital subtraction angiography; MRI, magnetic resonance imaging; and OR, odds ratio. *Most severely stenotic side. **Side with stenosis <50%. †No cut-off value presented.

Three studies could be included for the meta-analyses on the relation between sex and plaque ulceration (Figure 6A). The total number of patients was 3517 and all patients had stroke or transient ischemic attack. After pooling the effect estimates of these studies, we found an OR of 1.81 (95% CI, 1.30–2.51) for the presence of plaque ulceration in men.

We found a similar result for the presence of a thin-or-ruptured fibrous cap, although the OR resulting from the meta-analyses for thin-or-ruptured fibrous cap was higher (OR=2.98 [95% CI, 1.98–4.48]). For this analysis, we included 3 studies describing both symptomatic and asymptomatic participants with a total sample size of 1585 (Figure 6B).

Only the meta-analyses for thin-or-ruptured fibrous cap could be stratified for asymptomatic versus symptomatic populations (Figure S9). We found an OR of 4.94 (95% CI, 2.53–9.65) for the presence of thin-or-ruptured fibrous cap in the asymptomatic group, and an OR of 2.23 (95% CI, 1.35–3.68) in the symptomatic group (*P* value for subgroup differences=0.14).

### Plaque Inflammation

We found 3 studies reporting sex-stratified analyses for plaque inflammation. Strobl et al^45^ investigated the association of the target-to-background ratio in the carotid artery assessed with ^18^F-FDG positron emission tomography-computed tomography with several risk factors, showing that men have higher target-to-background ratio than women (unadjusted β=0.12 [95% CI, 0.06–0.18]). In line with this, Derlin et al^16^ reported that male oncologic patients more often had carotid radiotracer accumulation (*P*<0.0001). This study, however, did not show sex-stratified analyses for target-to-background ratio and standardized uptake values. Giannotti et al^20^ measured the average of the standardized uptake values across the whole plaque among patients with a recent ischemic stroke. They found no differences between men and women (mean standardized uptake values for men 1.77, for women 1.93, *P* value=0.39). Additional analyses for the hottest slice and the most diseased segment also showed no sex differences.

## Discussion

In this systematic review and meta-analysis, we demonstrate convincing evidence for differences between men and women in carotid atherosclerosis. All types of plaque features—plaque size, composition, and morphology—were larger or more common in men compared to women. Furthermore, we found sex differences in the amount of IPH, LRNC, and calcifications within the plaque.

### Sex Differences in Carotid Plaque Characteristics

First, we showed that all 3 measures of plaque size (1D, 2D, and 3D) were sex-dependent. Surprisingly, we found no sex differences in the normalized wall index. Since the normalized wall index is the ratio between the wall and total vessel size, it could be that the difference between men and women in plaque size is driven by differences in vessel size.^87^ On the contrary, the meta-analyses should be interpreted carefully since the heterogeneity of the studies was high. Variation in methods, such as measuring area versus volume or total wall versus plaque, could have provoked this heterogeneity and the contradictory results among the individual studies. Nevertheless, since the underlying pathophysiology of sex differences in plaque burden is important and vessel size indeed could vary between men and women, this topic deserves further investigation.

With regard to the quantitative aspects of specific plaque composition, we found that absolute but not relative volumes of calcifications are higher in men, and the exact opposite for LRNC. These results are 2 sides of the same coin: it appears that men have plaques with more IPH and LRNC and less calcifications, and women plaques with more calcifications and less IPH and LRNC, which might be in favor of women.

This study shows that sex differences exist in both symptomatic and asymptomatic populations. Among symptomatic patients, they are more pronounced for LRNC, stressing the importance to take sex into account in clinical work-ups. At the same time, we also have to be aware of sex differences in healthy or asymptomatic populations, which could be expressed in different lifetime risks of stroke.

A growing number of studies are performed on imaging of plaque inflammation. Since this is a rather new field of interest, little is known on differences between men and women, which is also demonstrated by our study. This lack of information is partly explained by multiple studies not stratifying for sex. To get more insight into sex-specific plaque pathophysiology, it is essential to take sex into account in new studies, starting at including adequately-sized samples of men and women.^88^ Furthermore, only 1 study that was included in this review was performed among stroke patients. The other 2 were performed in oncologic patients. Therefore, further research among selected patients with carotid atherosclerosis would also by highly valuable to make findings more generalizable to specific patient groups.

Sex differences in the coexistence of plaque characteristics requires further research. Indeed, we recently found that specific combinations, such as the presence of calcifications, LRNC, and IPH, are more frequently seen in male patients.^49^ This suggests that having a carotid plaque with multiple vulnerable plaque features could signify an even higher stroke risk and that new studies are needed to show whether these kind of plaques are indeed more prone to rupture.

### Mechanisms Underlying Sex Differences in Carotid Atherosclerosis

Evidence on possible explanations why men have more advanced plaques is emerging. Both sex and gender may contribute to a worse risk factor profile in men. However, the higher prevalence of modifiable risk factors as smoking, hypertension, and diabetes in men explains only the tip of the iceberg.^89^ Other explanations include genetic factors,^90^ sex hormones,^21^ and systemic inflammatory profiles.^91^ Gene activity is highly influenced by sex and varies over multiple tissues.^92,93^ For example, X- and Y-chromosomes affect the expression of genes located on the autosomes, leading to differences in gene expression and activity. Also sex steroids interfere with gene expression via multiple mechanisms such as interaction with epigenetic modifiers.^92^ Moreover, several studies point toward the protective role of estrogens on the premenopausal female vascular system,^94^ which is suggested to be lost in postmenopausal women.^21^ The gut microbiome could be an important mediator in this.^95^ Dysbiosis of the microbiome is associated with cardiovascular disease and atherosclerosis, probably via increasing the prevalence of traditional risk factors, but also by secretion of toxic metabolites. Besides sex hormones, also gender-specific dietary intake could affect the microbiome. Also sex differences in systematic inflammatory profiles may promote atherosclerosis.^89,91^ For instance, it has been reported that men have more circulating CD14^+^ and CD16^++^ monocytes, which have been associated with impaired endothelial function,^96^ intima-media thickness,^97^ and less carotid compliance,^98^ than women.^99^ Another mechanism could be differences in carotid anatomy, affecting hemodynamic parameters. For example, women tend to have larger outflow/inflow ratios. This means they have relatively larger outflow areas (internal and external carotid arteries) compared to the inflow area (common carotid artery), which affects the formation and distribution of carotid plaques. Low ratios, which are more seen in men, result in loss of flow energy, increasing local stress, and endothelial damage.^100^ More insight in these underlying mechanisms could contribute to the discovery of sex-specific therapeutic targets for atherosclerosis. This stresses, once again, the importance of including equal populations of male and female patients in studies and investigation of sex as an important component from animal models through clinical trials.^89^

### Clinical Implications and Future Directions

The found sex differences in carotid atherosclerosis are of clinically significant importance, since the composition of plaque affects the risk of (recurrent) stroke. Previous studies have shown that especially IPH contributes to a higher stroke risk.^101–103^ Carotid LRNC, calcifications, total plaque size, and plaque ulceration have also been reported as important risk factors.^5^ With regard to sex-specific risk prediction and treatment, it is essential to investigate the effect of these plaque characteristics per sex separately. We hypothesize that the stroke risk as a result of specific plaque compositions varies among men and women. This idea is supported by the recently published finding that especially in asymptomatic women, IPH increased the risk of atherosclerotic cardiovascular disease (hazard ratio=3.37 [95% CI, 1.81–6.25] for women versus hazard ratio=1.67 [95% CI, 0.98–2.79] for men).^53^ Studies on sex-specific risks for (recurrent) stroke risk related to carotid plaque composition are still lacking, probably due to low power, which stresses the importance of the inclusion of an adequate number of both men and women in clinical trials.

Summarizing, we conclude that men have more often vulnerable plaques than women. This has implications for the interpretation of carotid atherosclerosis in men and women during stroke workup, for instance. It is more likely that in men the plaques are more advanced including components like IPH. Currently, ultrasonography and computed tomography angiography are the most used modalities for carotid evaluation but these cannot reliably identify the presence of IPH.^104^ This underlines the relevance of using magnetic resonance imaging in the diagnostic workup, which is feasible in clinical practice since only 1 sequence is needed for the detection of IPH.^105^

It is important to realize that although the exact mechanisms of sex differences in carotid atherosclerosis are still unclear, we are already able to act on these differences. We can use this knowledge in clinical practice, being aware of differences in likelihood of having a vulnerable carotid plaque which affects patient’s stroke risk. Hence, the next step is to investigate the effect of plaque characteristics on stroke per sex separately. This will also allow us to make sex-specific risk scores in order to improve clinical decision making.

### Study Limitations

This meta-analysis has several limitations that deserve comment. First, we observed moderate to high heterogeneity among the included studies especially with regard to plaque size and carotid calcifications, probably due to differences in population characteristics such as history of cardiovascular disease and severity of stenosis. However, most individual studies reported strikingly consistent results.

We stratified the analyses for both asymptomatic versus symptomatic studies to underline sex differences. Unfortunately, for severity of atherosclerosis we could not stratify since only Ota et al^34^ reported on patients with carotid stenosis ≥50%. Most studies included patients with less severe carotid stenosis, did not select on carotid stenosis, or reported not explicitly on the severity.

In this meta-analysis, adjusting for potential confounders on the relation between sex and carotid atherosclerosis was not possible. We did not have individual patient data including both plaque and patient characteristics to perform these analyses. Since these confounders could yield insights in pathophysiology, it is important to adjust for several factors such as cardiovascular risk factors and vessel size in future studies.

In this review, we focused on the prevalence of carotid plaque characteristics rather than on explanations or treatment. These topics are also worth investigating and we have touched upon this in the discussion. We also decided to include only studies using modalities considered as golden standards nowadays for identification of plaque characteristics. Consequently, we may have missed interesting studies. However, this approach does makes our results more generalizable to clinical practice. Finally, we included sex as a criterion in the search strategy (see exact terms in the Supplemental Material). However, to limit the chance that we missed relevant articles that did not mention explicitly one of these terms in title or abstract, we also hand-searched the reference lists of the initially included articles.

### Conclusions

In this systematic review and meta-analysis, we demonstrate convincing evidence for sex differences in carotid atherosclerosis. All kinds of plaque features—plaque size, composition, and morphology—were more common or larger in men compared to women. Furthermore, we found sex differences in the amount of IPH, LRNC, and calcifications within the plaque. Our results highlight that sex is an important variable to include in both clinical-decision making and study designs. Further investigation of sex-specific stroke risks with regard to plaque composition is warranted.

## Article Information

### Acknowledgments

We thank Wichor M. Bramer‚ biomedical information specialist from the Medical Library of the Erasmus University Medical Center Rotterdam‚ for his assistance with designing the search query.

### Sources of Funding

None.

### Disclosures

None.

### Supplemental Material

Supplemental Methods

Tables S1–S3

Figures S1–S9

## Supplementary Material
