# Supplementary material for: Sex Differences in Carotid Atherosclerosis: A Systematic Review and Meta-Analysis
Source: Stroke. 2022 Nov 29;54(2):315–26. doi: 10.1161/STROKEAHA.122.041046 (PMC9855762; doi:10.1161/STROKEAHA.122.041046)
Supplement: Supplementary file 1 [file str-54-315-s001.pdf]

SUPPLEMENTAL MATERIAL

accompanying the article:

Sex differences in carotid atherosclerosis: a systematic review and meta-analyses

Dianne H.K. van Dam-Nolen MD, Nina C.M. van Egmond BSc,
Peter J. Koudstaal MD PhD, Aad van der Lugt MD PhD, Daniel Bos MD PhD

Supplemental methods

Search strategies

embase.com

((('atherosclerotic plaque'/exp OR atherosclerosis/de OR calcification/de OR 'artery calcification'/de OR 'brain calcification'/de OR 'blood vessel calcification'/de OR stenosis/de) AND ('carotid artery'/exp OR 'carotid sinus'/de OR 'carotid artery disease'/de)) OR 'carotid atherosclerosis'/de OR 'carotid artery obstruction'/exp OR (((carotid) NEAR/6 (plaque* OR atheroscler0* OR calci* OR stenosis OR occlusi*))):ab,ti) AND ('sex difference'/de OR 'sex ratio'/de OR 'sexual characteristics'/exp OR 'sex factor'/de OR (((sex* OR gender*) NEAR/3 (differen* OR depend* OR compar* OR predict* OR morpholog* OR composition* OR ratio OR distribution* OR characteristic* OR factor*)) OR ((men OR male OR man) NEAR/3 (woman OR women OR female) NEAR/6 (differen* OR depend* OR compar* OR vs OR predict* OR morpholog* OR composition* OR ratio OR distribution* OR characteristic* OR factor*))):ab,ti OR (gender* OR sex* OR ((men OR male OR man) NEAR/3 (woman OR women OR female))):ti) AND ('radiodiagnosis'/exp OR 'computed tomography scanner'/exp OR 'computer assisted tomography'/exp OR 'nuclear magnetic resonance imaging'/exp OR 'nuclear magnetic resonance scanner'/de OR (radiodiagnos* OR radiolog* OR radiogra* OR ((comput* OR positron*) NEAR/3 tomogra*) OR mri OR (magnet* NEAR/3 resonan*) OR cta OR ct OR pet OR ((cat) NEXT/1 scan*) OR angiogra* OR angioscintigra*):ab,ti) NOT ([animals]/lim NOT [humans]/lim) NOT ([Conference Abstract]/lim) AND [English]/lim

Medline Ovid

(((Plaque, Atherosclerotic/ OR Atherosclerosis/ OR Calcification, Physiologic/ OR Calcinosis/ OR Calcinosis/ OR Vascular Calcification/ OR stenosis/) AND (exp Carotid Artery/ OR Carotid Artery Diseases/)) OR Carotid Stenosis/ OR (((carotid) ADJ6 (plaque* OR atherosclero* OR calci* OR stenosis OR occlusi*))).ab,ti.) AND (Sex Characteristics/ OR exp Sex Distribution/ OR Sex Factors/ OR (((sex* OR gender*) ADJ3 (differen* OR depend* OR compar* OR predict* OR morpholog* OR composition* OR ratio OR distribution* OR characteristic* OR factor*)) OR ((men OR male OR man)

ADJ3 (woman OR women OR female) ADJ6 (differen* OR depend* OR compar* OR vs OR predict* OR morpholog* OR composition* OR ratio OR distribution* OR characteristic* OR factor*))).ab,ti. OR (gender* OR sex* OR ((men OR male OR man) ADJ3 (woman OR women OR female))).ti.) AND (exp Radiography/ OR exp Tomography/ OR (radiodiagnos* OR radiolog* OR radiogra* OR ((comput* OR positron*) ADJ3 tomogra*) OR mri OR (magnet* ADJ3 resonan*) OR cta OR ct OR pet OR ((cat) ADJ scan*) OR angiogra* OR angioscintigra*).ab,ti.) NOT (exp animals/ NOT humans/) AND english.la.

Web of science

(TS=((((carotid) NEAR/5 (plaque* OR atherosclero* OR calci* OR stenosis OR occlusi*)))) AND (TS= (((sex* OR gender*) NEAR/2 (differen* OR depend* OR compar* OR predict* OR morpholog* OR composition* OR ratio OR distribution* OR characteristic* OR factor*)) OR ((men OR male OR man) NEAR/2 (woman OR women OR female) NEAR/5 (differen* OR depend* OR compar* OR vs OR predict* OR morpholog* OR composition* OR ratio OR distribution* OR characteristic* OR factor*))) OR TI=(gender* OR sex* OR ((men OR male OR man) NEAR/2 (woman OR women OR female)))) AND TS=((radiodiagnos* OR radiolog* OR radiogra* OR ((comput* OR positron*) NEAR/2 tomogra*) OR mri OR (magnet* NEAR/2 resonan*) OR cta OR ct OR pet OR ((cat) NEAR/1 scan*) OR angiogra* OR angioscintigra*))) AND DT=(article) AND LA=(english)

Cochrane CENTRAL

((((carotid) NEAR/6 (plaque* OR atherosclero* OR calci* OR stenosis OR occlusi*))):ab,ti) AND ((((sex* OR gender*) NEAR/3 (differen* OR depend* OR compar* OR predict* OR morpholog* OR composition* OR ratio OR distribution* OR characteristic* OR factor*)) OR ((men OR male OR man) NEAR/3 (woman OR women OR female) NEAR/6 (differen* OR depend* OR compar* OR vs OR predict* OR morpholog* OR composition* OR ratio OR distribution* OR characteristic* OR factor*))):ab,ti OR (gender* OR sex* OR ((men OR male OR man) NEAR/3 (woman OR women OR female))):ti) AND ((radiodiagnos* OR radiolog* OR radiogra* OR ((comput* OR positron*) NEAR/3 tomogra*) OR mri OR (magnet* NEAR/3 resonan*) OR cta OR ct OR pet OR ((cat) NEXT/1 scan*) OR angiogra* OR angioscintigra*):ab,ti)

Google scholar

carotid plaque|atherosclerosis|calcification|stenosis|occlusion "sex|gender difference|comparison"|"men|male|man*woman|women|female" radiodiagnosis|radiology|radiography|"computed|computer tomography"|"magnetic resonance"|angiography

Assessment of risk of bias

A star is considered as 1 point. Per category (selection, information, and outcome) each study is classified to low, possible, or high risk of bias.

(1) Selection

1. Representativeness of the sample
   a. Truly representative of the average in the target population *
   b. Somewhat representative of the average in the target population *
   c. Selected group of users
   d. No description of the derivation of the cohort
2. Sample size
   a. Justified and satisfactory *
   b. Not justified
3. Method in case of selection based on carotid atherosclerosis:
   a. Cut-off value described (not relative to own cohort) *
   b. Cut-off value relative to own cohort (e.g. percentiles)
   c. Not described

Low risk of bias:	3 points (2 points if question 3 is not applicable)
Possible risk of bias:	1-2 points
High risk of bias:	0 points

(2) Information

1. Side included in the analyses:
   a. Per artery (one artery, or both arteries separately) **
   b. One artery (based on severity, e.g., greatest thickness) *
   c. Both arteries simultaneously *
   d. Not described
2. Symptomatic versus asymptomatic population:
   a. Either asymptomatic or symptomatic *
   b. Both asymptomatic and symptomatic (not separately analysed)
   c. Not described

Low risk of bias:	3 points
Possible risk of bias:	2 points
High risk of bias:	0-1 points

(3) Outcome

1. Used imaging modality for assessment of plaque characteristics:
   a. Golden standard (according to Table 1) *
   b. Multiple imaging modalities including no-golden standard options
   c. No-golden standard modality

2. Evaluation of plaque characteristics:
    a. Independent blind assessment *
    b. No independent blind assessment
    c. No description
3. Method used for evaluation of plaque characteristics:
    a. Manual / visual *
    b. Semi-automatic *
    c. Automatic
    d. No description

Low risk of bias:	3 points
Possible risk of bias:	1-2 points
High risk of bias:	0 points

Supplemental tables

Plaque characteristic	Ultrasound	DSA	CT(A)	MRI/MRA	PET
Calcifications	✗	✗	✓	✓	✗
Lipid-rich necrotic core	✗	✗	✗	✓	✗
Intraplaque haemorrhage	✗	✗	✗	✓	✗
Thin-or-ruptured fibrous cap	✗	✗	✗	✓	✗
Plaque ulceration	✗	✓	✓	✗	✗
Plaque inflammation	✗	✗	✗	✓	✓
Plaque size[1]	✗	✗	✓	✓	✗
Degree of stenosis	✗	✗	✓	✓	✗

Table S1. Overview of prerequisites regarding imaging modalities for specific plaque characteristics

[1] I.e., plaque thickness, area, volume, and normalized wall index. DSA = digital subtraction angiography; CT(A) = computed tomography (angiography); MRA = magnetic resonance angiography; MRI = magnetic resonance imaging; PET = positron emission tomography.

First author	Year	Study cohort name	Study design	Sample size	Men (n)	Mean age (years)	Plaque characteristics	Imaging modality	Included in meta-analyses	Reason not in meta-analyses
**Allison**[8]	2004	-	n.a.	650	357	57	Calcification presence; calcification score	CT	No	Overlap cohort DiTomasso
**Altaf**[68]	2007	-	n.a.	60	54		IPH presence	MRI	No	Overlap cohort Hosseini
**Altaf**[69]	2007	-	n.a.	66	46		IPH presence	MRI	No	Overlap cohort Hosseini
**Altaf**[70]	2008	-	n.a.	64	50		IPH presence	MRI	No	Overlap cohort Hosseini
**Altaf**[9]	2011	-	n.a.	51	28	72	IPH presence	MRI	No	Overlap cohort Hosseini
**Altaf**[71]	2013	-	n.a.	35	24		IPH presence	MRI	No	Overlap cohort Hosseini
**Altaf**[72]	2014	-	n.a.	123	85		IPH presence	MRI	No	Overlap cohort Hosseini
**Blake**[10]	2003	-	Clinical cohort + carotid stenosis	46	31	71	LRNC presence	MRI	Yes	-
**Bos**[11]	2015	Rotterdam Study	n.a.	2408	1147	70	Calcification volume	CT	No	Overlap cohort Van der Toorn (2020)
**Catalano**[12]	2021	MAGNETIC study	Clinical cohort, asymptomatic + carotid stenosis	260	198	71 (median)	Lumen volume; wall area	MRI	Yes	-
**Che**[13]	2021	-	Clinical cohort, symptomatic + carotid plaque	156	108	61	IPH presence	MRI	Yes	-
**Cheung**[14]	2011	-	Clinical cohort + carotid stenosis	217	109	70	IPH presence	MRI	Yes	-
**Den Brok**[15]	2020	-	Clinical cohort, symptomatic	883	487	70 (median)	Stenosis	CT/MRI	Yes	-
**Derlin**[16]	2011	-	n.a.	269	103	66	TBR, SUV_{max}, SHS	PET-CT	No	Insufficient studies
**DiTomasso**[17]	2010	-	Preventive medicine cohort	1160	640	57	Calcification presence	CT	Yes	-

**Divers**[73]	2010	DHS	n.a.	422	152	57	Calcification presence	CT	No	Overlap met Wagenknecht (2007) en Divers (2011)
**Divers**[74]	2011	DHS	Clinical cohort	753	303	56	Calcification presence	CT	Yes	-
**Eliasziw**[18]	1994	NASCET	Clinical trial, symptomatic + carotid stenosis	659	452	64-65	Ulceration presence	DSA	Yes	-
**Fanning**[19]	2006	-	n.a.	209	unknown	unknown	Calcification presence; calcification score	CT	No	No raw data reported
**Giannotti**[20]	2021	BIOVASC study	n.a.	25	18	65	SUV	PET-CT	No	Insufficient data
**Glisic**[21]	2018	Rotterdam Study	Population-based + carotid plaque	1480	835	64-66	IPH, LRNC, and calcification presence	MRI	Yes	-
**Gupta**[22]	2014	-	Clinical cohort + carotid stenosis	53	22	76-78	IPH presence	MRI	Yes	-
**Han**[23]	2020	CPC study	Clinical trial + carotid stenosis	182	104	54-58	LRNC presence; LRNC and calcification percentage; wall volume percentage; NWI	MRI	Yes	-
**Hosseini**[75]	2013	-	Clinical cohort, symptomatic + carotid stenosis	179	127	72	IPH presence	MRI	Yes	-
**Hosseini**[76]	2017	ICAD study	Clinical cohort, symptomatic + carotid stenosis	152	92	76-79	IPH presence	MRI	Yes	-
**Hyder**[24]	2007	-	n.a.	356	123	56	Calcification presence	CT	No	Overlap cohort DiTomasso
**Kandiyil**[25]	2012	-	n.a.	176	124	70-75	IPH presence	MRI	No	Overlap cohort Hosseini
**Kapral**[26]	2009	RCSN	Clinical cohort, symptomatic	5300	2809	75 (median)	Stenosis	CT/MRI/US	Yes	-
**Keenan**[27]	2009	-	n.a.	100		Stratified per decade	Lumen, wall, and total vessel volume; wall/outer wall ratio	MRI	No	Insufficient data
**Kume**[77]	2010	-	Clinical cohort + carotid stenosis	165	134	71	IPH presence	MRI	Yes	-
**Kurosaki**[78]	2011	-	Clinical cohort, symptomatic + carotid stenosis	62	50	76-79	IPH presence	MRI	Yes	-
**Larson**[28]	2020	-	Clinical cohort	643	352	60-76	IPH presence	MRI	Yes	-

**Liem**[29]	2015	PARISK study	n.a.	100	69	67-69	IPH presence	MRI	No	Overlap cohort Van Dam-Nolen (2022)
**Lovett**[30]	2003	ECST	Clinical trial, symptomatic + carotid stenosis	2672	1930	62-63	Ulceration presence	DSA	Yes	-
**McLaughlin**[31]	2015	-	Clinical trial, symptomatic + carotid stenosis	726	387	64	IPH presence; ulceration presence; plaque thickness; stenosis	MRI	Only IPH	Insufficient data
**McNally**[79]	2012	-	Clinical cohort	266	127	66-76	IPH presence	MRI	Yes	-
**Noguchi**[80]	2011	-	Clinical cohort	217	189	68-69	IPH presence	MRI	Yes	-
**Odink**[32]	2007	Rotterdam Study	n.a.	600	314	74	Calcification presence; calcification volume; calcification score	CT	No	Overlap cohort Van der Toorn (2020)
**Odink**[33]	2010	Rotterdam Study	n.a.	1003	485	71-72	Calcification presence; calcification score	CT	No	Overlap cohort Van der Toorn (2020)
**Ota**[34]	2010	-	Clinical cohort, asymptomatic + carotid stenosis	131	67	69-70	IPH, LRNC, and calcification presence; IPH, LRNC, and calcification percentage; TRFC presence; stenosis	MRI	Yes	-
**Ota**[35]	2013	-	Clinical cohort, asymptomatic + carotid stenosis	96	50	unknown	IPH, LRNC, and calcification presence; IPH, LRNC, and calcification percentage; TRFC presence	MRI	Yes	-
**Pletsch-Borba**[81]	2017	Rotterdam Study	n.a.	198	113	68	IPH, LRNC, and calcification presence	MRI	No	Overlap cohort Glisic (2018)
**Qiao**[36]	2012	-		47	36	73	IPH presence	MRI	Yes	-
**Register**[37]	2013	-	n.a.	479	207	56	Calcification presence	CT	No	Overlap cohort Divers (2011)
**Register**[38]	2014	-	n.a.	450	197	55	Calcification presence	CT	No	Overlap cohort Divers (2011)
**Rose**[39]	2016	-	n.a.	68	38	45-47	Lumen, wall, and total vessel volume; wall/outer wall ratio	MRI	No	Insufficient data
**Sakamoto**[40]	2010	-	n.a.	30	28	72	IPH and LRNC presence	MRI	No	Insufficient data
**Scheffler**[41]	2021	-	Clinical cohort, asymptomatic + carotid stenosis	62	36	74	IPH presence	MRI	Yes	-

**Selwaness**[82]	2014	Rotterdam Study	n.a.	1414	749	72	IPH, LRNC and calcification presence; wall thickness; stenosis	MRI	No	Overlap Glisic (2018) and Van den Bouwhuijsen (2012)
**Selwaness**[42]	2016	Rotterdam Study	Population-based cohort, asymptomatic + carotid plaque	1562	839	73	Lumen volume; NWI	MRI	Yes	-
**Singh**[83]	2013	-	n.a.	216	103	68	IPH presence	MRI	No	Overlap cohort Singh (2017)
**Singh**[43]	2017	-	Clinical cohort, symptomatic + carotid stenosis	906	420	67	IPH presence	MRI	Yes	-
**Song**[44]	2021	-	Clinical cohort, symptomatic	189	92	65-78	IPH, LRNC, and calcification volume	CT	Yes	-
**Strobl**[45]	2013	-	n.a.	315	123	58	TBR	PET-CT	No	Insufficient studies
**Sun**[84]	2016	-		176	123	70	IPH presence	MRI	Yes	-
**Turc**[85]	2012	HIRISC	Clinical cohort + carotid stenosis	234	179	68-71	IPH presence	MRI	Yes	-
**Uehara**[46]	1996	-	n.a.	67	49	60	Stenosis	MRI	No	Other cut-off value
**Underhill**[47]	2008	CAMPS	Clinical cohort	191	95	58-61	LRNC and calcification presence; lumen, total vessel, and wall area; plaque thickness; NWI	MRI	Yes	-
**Van Dam-Nolen**[48]	2021	PARISK study	n.a.	224	156	69	IPH, LRNC, and calcification presence; IPH, LRNC, and calcification volume; ulceration and TRFC presence; plaque area; stenosis	CT/MRI	No	Overlap cohort Van Dam-Nolen (2022), insufficient data for plaque area
**Van Dam-Nolen**[49]	2022	PARISK study	Clinical cohort, symptomatic + carotid stenosis	182	136	68	IPH, LRNC, and calcification presence; IPH, LRNC, and calcification volume; ulceration and TRFC presence; plaque volume; stenosis	CT/MRI	Yes	-

**Van den Bouwhuijsen**[50]	2012	Rotterdam Study	Population-based cohort, asymptomatic + carotid plaque	1866	975	70	IPH, LRNC, and calcification presence; wall thickness; stenosis	MRI	Only wall thickness	Overlap cohort Glisic (2018)
**Van der Toorn**[51]	2020	Rotterdam Study	Population-based cohort	2357	1118	69	Calcification presence; calcification volume	CT	Yes	-
**Van der Toorn**[52]	2021	Rotterdam Study	n.a.	2167	983	69	Calcification presence; calcification volume	CT	No	Overlap cohort Van der Toorn (2020)
**Van der Toorn**[53]	2022	Rotterdam Study	n.a.	1349	681	72	IPH, LRNC, and calcification presence; plaque thickness; stenosis	MRI	No	Overlap cohort Van den Bouwhuijsen (2012) / Glisic (2018)
**Van Gils**[54]	2013	-	Clinical cohort, symptomatic + carotid plaque	222	141	61	Calcification volume	CT	Yes	-
**Van Velzen**[55]	2021	PASS	n.a.	1480	961	73	Stenosis	CT/MRI/US	No	Other cut-off value
**Voigt**[56]	2020	DUST	Clinical cohort	1397	797	67	Stenosis	CT	Yes	-
**Volcik**[57]	2010	ARIC study	n.a.	1701	870	70	LRNC presence; LRNC volume; plaque thickness; plaque volume; cap thickness	MRI	No	Overlap cohort Wagenknecht (2009)
**Vukadinovic**[58]	2012	-	n.a.	90	57	67	LRNC and calcification percentage; plaque volume	CT	No	Insufficient data
**Wagenknecht**[59]	2007	DHS	Clinical cohort	1112	502	62	Calcification presence	CT	Yes	-
**Wagenknecht**[60]	2009	ARIC study	Population-based cohort, + carotid plaque	1769	861	70	LRNC presence; LRNC volume; plaque thickness; plaque volume; cap thickness	MRI	Yes	-
**Wasserman**[61]	2008	MESA	Population-based cohort, asymptomatic + carotid plaque	214	121	67	LRNC presence	MRI	Yes	-
**Wasserman**[62]	2010	ARIC study	n.a.	1769	761	71	LRNC presence; LRNC volume; plaque thickness; plaque volume; cap thickness	MRI	No	Overlap cohort Wagenknecht (2009)
**Yamada**[63]	2018	-	Clinical cohort + carotid stenosis	152	115	78-79	IPH presence	MRI	Yes	-
**Yoshimura**[86]	2011	-	Clinical cohort + carotid stenosis	112	96	70-71	IPH presence	MRI	Yes	-

**Yuan[64]**	2016	DHS	n.a.	1315	552	56-63	Calcification presence	CT	No	Overlap cohort Wagenknecht (2007)
**Zhang[65]**	2015	-	Clinical cohort, symptomatic	860	599	62	Calcification presence; stenosis	CT	Yes	-
**Zhang[66]**	2021	CARE-II	Clinical cohort, symptomatic + carotid plaque	567	404	62	IPH, LRNC, and calcification presence; TRFC presence; plaque thickness, plaque area; NWI	MRI	Yes	-
**Zhao[67]**	2014	AIM-HIGH	Clinical trial	214	175	61	LRNC presence	MRI	Yes	-

Table S2. Overview of studies included in the review and selection for meta-analyses

AIM-HIGH = Atherothrombosis Intervention in Metabolic Syndrome With Low HDL/High Triglycerides; ARIC = Atherosclerosis Risk in Communities; BIOVASC = Biomarkers Imaging Vulnerable Atherosclerosis in Symptomatic Carotid disease; CAMPS = Carotid Atherosclerosis MRI Progression Study; CARE = Chinese Atherosclerosis Risk Evaluation; CPC = Carotid Plaque Composition; CT = computed tomography; DHS = Diabetes Heart Study; DSA = digital subtraction angiography; DUST = Dutch Acute Stroke Trial; ECST = European Carotid Surgery Trial; HIRISC = High-Resolution magnetic resonance Imaging in atherosclerotic Stenosis of the Carotid artery; ICAD = Imaging in Carotid Artery Disease; IPH = intraplaque hemorrhage; LRNC = lipid-rich necrotic core; MAGNETIC = Magnetic resonance imaging As a Gold standard for Noninvasive Evaluation of Atherosclerotic Involvement of Carotid arteries; MESA = Multi-Ethnic Study of Atherosclerosis; MRI = magnetic resonance imaging; NASCET = North American Symptomatic Carotid Endarterectomy Trial; NWI = normalized wall index; PARISK = Plaque At RISK; PASS = Preventive Antibiotics in Stroke Study; PET = positron emission tomography; RCSN = Registry of the Canadian Stroke Network; SHS = single hottest slice; SUV = standardized uptake value; TBR = target-to-background ratio; TRFC = thin-or-ruptured fibrous cap; US = ultrasound

		Selection				Information			Outcome			
1st author	year	Representativeness of the exposed cohort	Sample size	Method carotid atherosclerosis cut-off	Score	Side included	Symptomatic versus asymptomatic	Score	Used imaging modality	Evaluation of plaque characteristics	Method for evaluation	Score
Blake	2003	b. Somewhat representative of the average in the target population *	b. Not justified	a. Cut-off value described (not relative to own cohort) *	Possible	c. Both arteries simultaneously *	b. Both asymptomatic and symptomatic (not separately analysed)	High	a. Golden standard (according to Table 1) *	a. Independent blind assessment *	a. Manual / visual *	Low
Bouwhuijsen	2011	b. Somewhat representative of the average in the target population *	a. Justified and satisfactory *	a. Cut-off value described (not relative to own cohort) *	Low	c. Both arteries simultaneously *	a. Either asymptomatic or symptomatic *	Possible	a. Golden standard (according to Table 1) *	a. Independent blind assessment *	a. Manual / visual *	Low
Brok	2020	a. Truly representative of the average in the target population *	a. Justified and satisfactory *	NA	Low	d. Not described	a. Either asymptomatic or symptomatic *	High	b. Multiple imaging modalities including no-golden standard options	b. No independent blind assessment	a. Manual / visual *	Possible
Catalano	2021	c. Selected group of users	a. Justified and satisfactory *	a. Cut-off value described (not relative to own cohort) *	Possible	d. Not described	a. Either asymptomatic or symptomatic *	High	a. Golden standard (according to Table 1) *	c. No description	d. No description	Possible
Che	2021	b. Somewhat representative of the average in the target population *	a. Justified and satisfactory *	a. Cut-off value described (not relative to own cohort) *	Low	c. Both arteries simultaneously *	a. Either asymptomatic or symptomatic *	Possible	a. Golden standard (according to Table 1) *	a. Independent blind assessment *	b. Semi-automatic *	Low
Cheung	2011	c. Selected group of users	a. Justified and satisfactory *	a. Cut-off value described (not relative to own cohort) *	Possible	c. Both arteries simultaneously *	a. Either asymptomatic or symptomatic *	Possible	a. Golden standard (according to Table 1) *	a. Independent blind assessment *	a. Manual / visual *	Low
Dam-Nolen	2022	b. Somewhat representative of the average in the target population *	a. Justified and satisfactory *	a. Cut-off value described (not relative to own cohort) *	Low	a. Per artery (one artery, or both arteries separately) **	a. Either asymptomatic or symptomatic *	Low	a. Golden standard (according to Table 1) *	a. Independent blind assessment *	a. Manual / visual *	Low
DiTomasso	2010	b. Somewhat representative of the average in the target population *	a. Justified and satisfactory *	NA	Low	c. Both arteries simultaneously *	b. Both asymptomatic and symptomatic (not separately analysed)	High	a. Golden standard (according to Table 1) *	c. No description	b. Semi-automatic *	Possible
Divers	2011	b. Somewhat representative of the average in the target population *	a. Justified and satisfactory *	NA	Low	c. Both arteries simultaneously *	b. Both asymptomatic and symptomatic (not separately analysed)	High	a. Golden standard (according to Table 1) *	c. No description	b. Semi-automatic *	Possible
Eliasziw	1994	b. Somewhat representative of the average in the target population *	a. Justified and satisfactory *	a. Cut-off value described (not relative to own cohort) *	Low	a. Per artery (one artery, or both arteries separately) **	a. Either asymptomatic or symptomatic *	Low	a. Golden standard (according to Table 1) *	a. Independent blind assessment *	a. Manual / visual *	Low
Gils	2013	b. Somewhat representative of the average in the target population *	a. Justified and satisfactory *	c. Not described	Possible	c. Both arteries simultaneously *	a. Either asymptomatic or symptomatic *	Possible	a. Golden standard (according to Table 1) *	a. Independent blind assessment *	b. Semi-automatic *	Low
Glisic	2018	b. Somewhat representative of the average in the target population *	a. Justified and satisfactory *	a. Cut-off value described (not relative to own cohort) *	Low	c. Both arteries simultaneously *	a. Either asymptomatic or symptomatic *	Possible	a. Golden standard (according to Table 1) *	a. Independent blind assessment *	a. Manual / visual *	Low
Gupta	2014	b. Somewhat representative of the average in the target population *	b. Not justified	a. Cut-off value described (not relative to own cohort) *	Possible	c. Both arteries simultaneously *	c. Not described	High	a. Golden standard (according to Table 1) *	a. Independent blind assessment *	a. Manual / visual *	Low
Han	2020	b. Somewhat representative of the average in the target population *	a. Justified and satisfactory *	a. Cut-off value described (not relative to own cohort) *	Low	b. One artery (based on severity, e.g., greatest thickness) *	a. Either asymptomatic or symptomatic *	Possible	a. Golden standard (according to Table 1) *	a. Independent blind assessment *	b. Semi-automatic *	Low
Hosseini	2013	b. Somewhat representative of the average in the target population *	a. Justified and satisfactory *	a. Cut-off value described (not relative to own cohort) *	Low	a. Per artery (one artery, or both arteries separately) **	a. Either asymptomatic or symptomatic *	Low	a. Golden standard (according to Table 1) *	a. Independent blind assessment *	a. Manual / visual *	Low
Hosseini	2017	c. Selected group of users	a. Justified and satisfactory *	a. Cut-off value described (not relative to own cohort) *	Possible	a. Per artery (one artery, or both arteries separately) **	a. Either asymptomatic or symptomatic *	Low	a. Golden standard (according to Table 1) *	a. Independent blind assessment *	b. Semi-automatic *	Low
Kandiyil	2012	b. Somewhat representative of the average in the target population *	a. Justified and satisfactory *	a. Cut-off value described (not relative to own cohort) *	Low	a. Per artery (one artery, or both arteries separately) **	a. Either asymptomatic or symptomatic *	Low	a. Golden standard (according to Table 1) *	a. Independent blind assessment *	a. Manual / visual *	Low
Kapral	2009	b. Somewhat representative of the average in the target population *	a. Justified and satisfactory *	NA	Low	b. One artery (based on severity, e.g., greatest thickness) *	a. Either asymptomatic or symptomatic *	Low	b. Multiple imaging modalities including no-	c. No description	d. No description	High

									golden standard options			
Kume	2010	c. Selected group of users	a. Justified and satisfactory *	a. Cut-off value described (not relative to own cohort) *	Possible	d. Not described	b. Both asymptomatic and symptomatic (not separately analysed)	High	a. Golden standard (according to Table 1) *	a. Independent blind assessment *	a. Manual / visual *	Low
Kurosaki	2011	c. Selected group of users	a. Justified and satisfactory *	a. Cut-off value described (not relative to own cohort) *	Possible	d. Not described	a. Either asymptomatic or symptomatic *	High	a. Golden standard (according to Table 1) *	c. No description	a. Manual / visual *	Possible
Larson	2020	c. Selected group of users	a. Justified and satisfactory *	NA	Possible	c. Both arteries simultaneously *	b. Both asymptomatic and symptomatic (not separately analysed)	High	a. Golden standard (according to Table 1) *	b. No independent blind assessment	a. Manual / visual *	Low
Lovett	2003	b. Somewhat representative of the average in the target population *	a. Justified and satisfactory *	c. Not described	Possible	a. Per artery (one artery, or both arteries separately) **	a. Either asymptomatic or symptomatic *	Low	a. Golden standard (according to Table 1) *	c. No description	a. Manual / visual *	Possible
McLaughlin	2015	b. Somewhat representative of the average in the target population *	a. Justified and satisfactory *	c. Not described	Possible	a. Per artery (one artery, or both arteries separately) **	a. Either asymptomatic or symptomatic *	Low	a. Golden standard (according to Table 1) *	a. Independent blind assessment *	a. Manual / visual *	Low
McNally	2012	b. Somewhat representative of the average in the target population *	a. Justified and satisfactory *	NA	Low	a. Per artery (one artery, or both arteries separately) **	b. Both asymptomatic and symptomatic (not separately analysed)	Possible	a. Golden standard (according to Table 1) *	c. No description	a. Manual / visual *	Possible
Noguchi	2011	b. Somewhat representative of the average in the target population *	a. Justified and satisfactory *	NA	Low	c. Both arteries simultaneously *	b. Both asymptomatic and symptomatic (not separately analysed)	High	a. Golden standard (according to Table 1) *	c. No description	a. Manual / visual *	Possible
Ota	2010	c. Selected group of users	a. Justified and satisfactory * (but not for volumes)	a. Cut-off value described (not relative to own cohort) *	Possible	b. One artery (based on severity, e.g., greatest thickness) *	a. Either asymptomatic or symptomatic *	Possible	a. Golden standard (according to Table 1) *	a. Independent blind assessment *	a. Manual / visual *	Low
Ota	2013	c. Selected group of users	a. Justified and satisfactory * (but not for volumes)	a. Cut-off value described (not relative to own cohort) *	Possible	b. One artery (based on severity, e.g., greatest thickness) *	a. Either asymptomatic or symptomatic *	Possible	a. Golden standard (according to Table 1) *	a. Independent blind assessment *	a. Manual / visual *	Low
Qiao	2012	c. Selected group of users	b. Not justified	a. Cut-off value described (not relative to own cohort) *	Possible	c. Both arteries simultaneously *	b. Both asymptomatic and symptomatic (not separately analysed)	High	a. Golden standard (according to Table 1) *	a. Independent blind assessment *	b. Semi-automatic *	Low
Register	2013	b. Somewhat representative of the average in the target population *	a. Justified and satisfactory *	NA	Low	c. Both arteries simultaneously *	b. Both asymptomatic and symptomatic (not separately analysed)	High	a. Golden standard (according to Table 1) *	c. No description	b. Semi-automatic *	Possible
Scheffler	2021	b. Somewhat representative of the average in the target population *	a. Justified and satisfactory *	c. Not described	Possible	c. Both arteries simultaneously *	a. Either asymptomatic or symptomatic *	Possible	a. Golden standard (according to Table 1) *	a. Independent blind assessment *	b. Semi-automatic *	Low
Selwaness	2016	b. Somewhat representative of the average in the target population *	a. Justified and satisfactory *	a. Cut-off value described (not relative to own cohort) *	Low	c. Both arteries simultaneously *	a. Either asymptomatic or symptomatic *	Possible	a. Golden standard (according to Table 1) *	c. No description	c. Automatic	High
Singh	2017	b. Somewhat representative of the average in the target population *	a. Justified and satisfactory *	a. Cut-off value described (not relative to own cohort) *	Low	c. Both arteries simultaneously *	a. Either asymptomatic or symptomatic *	Possible	a. Golden standard (according to Table 1) *	c. No description	a. Manual / visual *	Possible
Song	2021	b. Somewhat representative of the average in the target population *	a. Justified and satisfactory *	NA	Low	a. Per artery (one artery, or both arteries separately) **	a. Either asymptomatic or symptomatic *	Low	c. No-golden standard modality (for IPH and LRNC)	a. Independent blind assessment *	b. Semi-automatic *	Possible
Sun	2016	b. Somewhat representative of the average in the target population *	a. Justified and satisfactory *	a. Cut-off value described (not relative to own cohort) *	Low	a. Per artery (one artery, or both arteries separately) **	a. Either asymptomatic or symptomatic *	Low	a. Golden standard (according to Table 1) *	c. No description	a. Manual / visual *	Possible
Toorn	2020	b. Somewhat representative of the average in the target population *	a. Justified and satisfactory *	NA	Low	c. Both arteries simultaneously *	b. Both asymptomatic and symptomatic (not separately analysed)	High	a. Golden standard (according to Table 1) *	c. No description	b. Semi-automatic *	Possible
Toorn	2020	b. Somewhat representative of the average in the target population *	a. Justified and satisfactory *	NA	Low	c. Both arteries simultaneously *	b. Both asymptomatic and symptomatic (not separately analysed)	High	a. Golden standard (according to Table 1) *	c. No description	b. Semi-automatic *	Possible

Underhill	2008	b. Somewhat representative of the average in the target population *	a. Justified and satisfactory *	NA	Low	c. Both arteries simultaneously *	c. Not described	High	a. Golden standard (according to Table 1) *	a. Independent blind assessment *	a. Manual / visual *	Low
Voigt	2020	b. Somewhat representative of the average in the target population *	a. Justified and satisfactory *	NA	Low	c. Both arteries simultaneously *	a. Either asymptomatic or symptomatic *	Possible	a. Golden standard (according to Table 1) *	c. No description	a. Manual / visual *	Possible
Wagenknecht	2007	b. Somewhat representative of the average in the target population *	a. Justified and satisfactory *	NA	Low	c. Both arteries simultaneously *	b. Both asymptomatic and symptomatic (not separately analysed)	High	a. Golden standard (according to Table 1) *	c. No description	b. Semi-automatic *	Possible
Wagenknecht	2009	b. Somewhat representative of the average in the target population *	a. Justified and satisfactory *	b. Cut-off value relative to own cohort (e.g. percentiles)	Possible	d. Not described	b. Both asymptomatic and symptomatic (not separately analysed)	High	a. Golden standard (according to Table 1) *	c. No description	a. Manual / visual *	Possible
Wasserman	2008	b. Somewhat representative of the average in the target population *	a. Justified and satisfactory *	b. Cut-off value relative to own cohort (e.g. percentiles)	Possible	b. One artery (based on severity, e.g., greatest thickness) *	a. Either asymptomatic or symptomatic *	Possible	a. Golden standard (according to Table 1) *	a. Independent blind assessment *	a. Manual / visual *	Low
Yamada	2018	c. Selected group of users	a. Justified and satisfactory *	a. Cut-off value described (not relative to own cohort) *	Possible	a. Per artery (one artery, or both arteries separately) **	b. Both asymptomatic and symptomatic (not separately analysed)	Possible	a. Golden standard (according to Table 1) *	a. Independent blind assessment *	a. Manual / visual *	Low
Yoshimura	2011	c. Selected group of users	a. Justified and satisfactory *	NA	Possible	b. One artery (based on severity, e.g., greatest thickness) *	b. Both asymptomatic and symptomatic (not separately analysed)	High	a. Golden standard (according to Table 1) *	c. No description	d. No description	Possible
Zhang	2015	b. Somewhat representative of the average in the target population *	a. Justified and satisfactory *	NA	Low	c. Both arteries simultaneously *	a. Either asymptomatic or symptomatic *	Possible	a. Golden standard (according to Table 1) *	a. Independent blind assessment *	a. Manual / visual *	Low
Zhang	2021	b. Somewhat representative of the average in the target population *	a. Justified and satisfactory *	a. Cut-off value described (not relative to own cohort) *	Low	a. Per artery (one artery, or both arteries separately) **	a. Either asymptomatic or symptomatic *	Low	a. Golden standard (according to Table 1) *	a. Independent blind assessment *	a. Manual / visual *	Low
Zhao	2014	c. Selected group of users	a. Justified and satisfactory *	NA	Possible	b. One artery (based on severity, e.g., greatest thickness) *	b. Both asymptomatic and symptomatic (not separately analysed)	High	a. Golden standard (according to Table 1) *	a. Independent blind assessment *	b. Semi-automatic *	Low

Table S3. Assessment of risk of bias per study.

Each study is evaluated for risk of bias per domain (selection, information, and outcome) and per domain classified for low, possible, or high risk of bias.

Supplemental figures

Study	Study population	Modality	Side	Beta	95% CI	Men	Women
Underhill (2008)	With CAD, assessed at ICA level	MRI	Average	-0.23	[-0.63; 0.17]	49	48
Underhill (2008)	Without CAD, assessed at ICA level	MRI	Average	-0.15	[-0.55; 0.26]	46	48
Selwaness (2016)	Asymptomatic + carotid plaque ≥2.5 mm	MRI	Both	-0.19	[-0.29; -0.09]	839	723
Han (2020)	With CAD or carotid stenosis (15-69%)	MRI	One artery *	-0.14	[-0.43; 0.15]	104	78
Zhang (2021)	Symptomatic + carotid plaque ≥1.5 mm	MRI	Ipsilateral	0.35	[0.16; 0.53]	404	163
Zhang (2021)	Symptomatic + carotid plaque ≥1.5 mm	MRI	Contralateral	0.33	[0.15; 0.51]	404	163
**Random effects model**				**0.02**	**[-0.21; 0.25]**	**1846**	**1223**

Heterogeneity: $I^2 = 88\%$, $p < 0.01$

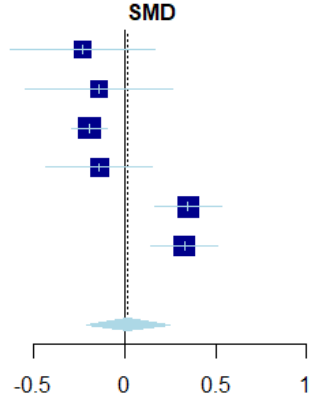

Figure S1. Meta-analyses on the association between sex and normalized wall index

The size of the box which represents the beta, is proportional to the weight of the study. The diamond is the result of the random-effect meta-analysis. Abbreviations: CAD = coronary artery disease; CI = confidence interval; ICA = internal carotid artery; MRI = magnetic resonance imaging; SMD = standardized mean difference.

A. Degree of stenosis 50-69%

Study	Study population	Modality	Side	OR	95% CI	Men	Women
Kapral (2009)	Symptomatic	Doppler/US/CTA/MRA/DSA	One artery*	0.89	[0.74; 1.07]	2809	2491
Brok (2020)	Symptomatic	Duplex/CTA/MRA	Unknown	2.14	[1.05; 4.34]	487	396
**Random effects model**				**1.28**	**[0.55; 3.00]**	**3296**	**2887**

Heterogeneity: $I^2 = 82\%$, $p = 0.02$

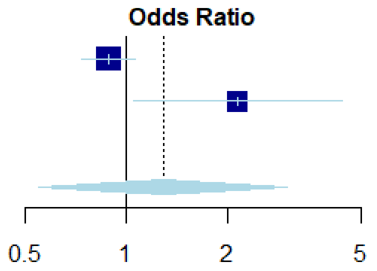

B. Degree of stenosis 70-99%

Study	Study population	Modality	Side	OR	95% CI	Men	Women
Kapral (2009)	Symptomatic	Doppler/US/CTA/MRA/DSA	One artery*	1.40	[1.14; 1.71]	2809	2491
Brok (2020)	Symptomatic	Duplex/CTA/MRA	Unknown	2.37	[1.38; 4.08]	487	396
Voigt (2020)	Symptomatic	CTA	Any	1.82	[1.40; 2.38]	797	600
**Random effects model**				**1.69**	**[1.30; 2.21]**	**4093**	**3487**

Heterogeneity: $I^2 = 57\%$, $p = 0.10$

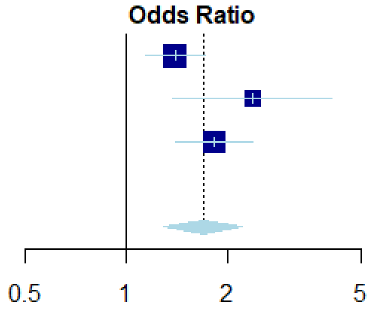

Figure S2. Meta-analyses on the association between sex and stenosis degree

The size of the box which represents the odds ratio, is proportional to the weight of the study. The diamond is the result of the random-effect meta-analysis. Abbreviations: CI = confidence interval; CTA = computed tomography angiography; DSA = digital subtraction angiography; MRA = magnetic resonance angiography; OR = odds ratio; US = ultrasound. * = most severely stenotic side

A. Absolute volume of calcifications

Study	Study population description	Modality	Side	Beta	95% CI	Men	Women
Gils (2013)	Symptomatic + carotid plaque †	CT	Average of both sides	0.31	[0.03; 0.58]	141	81
Toorn (2020)	Randomly selected from population	CT	Both sides	0.41	[0.33; 0.50]	1118	1239
Song (2021)	Symptomatic + ESUS	CT	Ipsilateral	0.53	[0.11; 0.94]	42	52
Song (2021)	Symptomatic + ESUS	CT	Contralateral	0.33	[-0.08; 0.74]	42	52
Song (2021)	Symptomatic + AF	CT	Ipsilateral	0.67	[0.26; 1.09]	50	45
Song (2021)	Symptomatic + AF	CT	Contralateral	0.16	[-0.24; 0.57]	50	45
Dam-Nolen (2022)	Symptomatic + carotid stenosis <70%	CT	Ipsilateral	0.04	[-0.30; 0.38]	122	47
**Random effects model**				**0.37**	**[0.25; 0.48]**	**1565**	**1561**

Heterogeneity: I^2 = 28%, p = 0.22

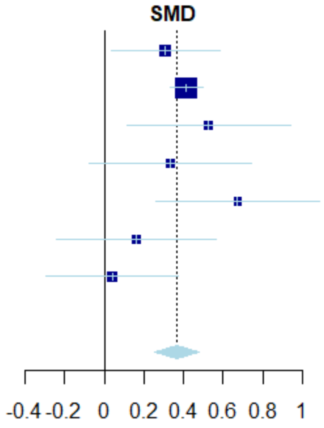

B. Relative volume of calcifications

Study	Study population	Modality	Side	Beta	95% CI	Men	Women
Ota (2010)	Asymptomatic + carotid stenosis ≥50%	MRI	One artery *	-0.00	[-0.38; 0.37]	56	55
Ota (2013)	Asymptomatic + carotid stenosis <50%	MRI	One artery **	0.14	[-0.32; 0.60]	43	32
Han (2020)	With CAD or carotid stenosis (15-69%)	MRI	One artery ***	0.00	[-0.29; 0.29]	104	78
Zhang (2021)	Symptomatic + carotid plaque ≥1.5 mm	MRI	Ipsilateral	-0.16	[-0.35; 0.02]	404	163
Zhang (2021)	Symptomatic + carotid plaque ≥1.5 mm	MRI	Contralateral	0.14	[-0.04; 0.33]	404	163
**Random effects model**				**0.01**	**[-0.15; 0.16]**	**1011**	**491**

Heterogeneity: I^2 = 32%, p = 0.21

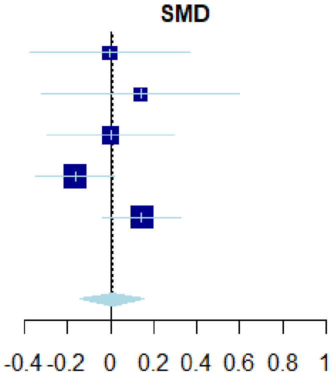

Figure S3. Meta-analyses on the association between sex and amount of carotid calcifications

The size of the box which represents the beta, is proportional to the weight of the study. The diamond is the result of the random-effect meta-analysis. Abbreviations: AF = atrial fibrillation; CAD = coronary artery disease; CI = confidence interval; CT = computed tomography; DM = diabetes mellitus; ESUS = embolic stroke of undetermined origin; MRI = magnetic resonance imaging; SMD = standardized mean difference. * = most sevesrely stenotic side; ** = side with stenosis <50%; *** = only assessed on MRI slices including lipid. † No cut-off value presented.

A. Absolute volume of lipid-rich necrotic copre

Study	Study population	Modality	Side	Beta	95% CI	Men	Women
Wagenknecht (2009)	African American + carotid plaque ≥1 or 1.28 mm	MRI	Unknown	0.16	[-0.24; 0.56]	44	54
Wagenknecht (2009)	White + carotid plaque ≥1 or 1.28 mm	MRI	Unknown	-0.23	[-0.42; -0.05]	281	190
Song (2021)	Symptomatic + ESUS	CT	Ipsilateral	0.08	[-0.32; 0.49]	42	52
Song (2021)	Symptomatic + ESUS	CT	Contralateral	0.01	[-0.40; 0.41]	42	52
Song (2021)	Symptomatic + AF	CT	Ipsilateral	0.25	[-0.15; 0.65]	50	45
Song (2021)	Symptomatic + AF	CT	Contralateral	-0.29	[-0.69; 0.12]	50	45
Dam-Nolen (2022)	Symptomatic + carotid stenosis <70%	MRI	Ipsilateral	0.54	[0.12; 0.95]	114	28
**Random effects model**				**0.05**	**[-0.17; 0.26]**	**623**	**466**

Heterogeneity: $I^2 = 63\%$, $p = 0.01$

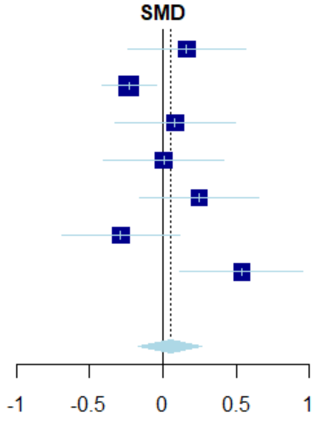

B. Relative volume of lipid-rich necrotic core

Study	Study population	Modality	Side	Beta	95% CI	Men	Women
Ota (2010)	Asymptomatic + carotid stenosis ≥50%	MRI	One artery *	0.58	[0.13; 1.04]	49	32
Ota (2013)	Asymptomatic + carotid stenosis <50%	MRI	One artery **	0.79	[0.15; 1.43]	25	17
Han (2020)	With CAD or carotid stenosis (15-69%)	MRI	One artery ***	0.48	[0.20; 0.77]	104	96
Zhang (2021)	Symptomatic + carotid plaque ≥1.5 mm	MRI	Ipsilateral	0.33	[0.14; 0.51]	404	163
Zhang (2021)	Symptomatic + carotid plaque ≥1.5 mm	MRI	Contralateral	0.43	[0.24; 0.61]	404	163
**Random effects model**				**0.42**	**[0.31; 0.53]**	**986**	**471**

Heterogeneity: $I^2 = 0\%$, $p = 0.56$

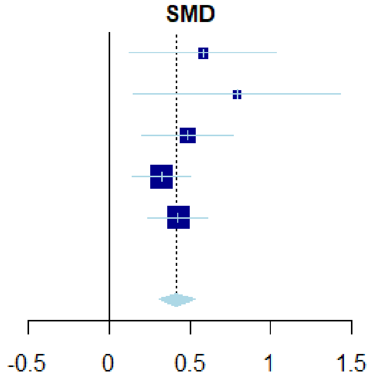

Figure S4. Meta-analyses on the association between sex and amount of lipid-rich necrotic core

The size of the box which represents the beta, is proportional to the weight of the study. The diamond is the result of the random-effect meta-analysis. Abbreviations: AF = atrial fibrillation; CAD = coronary artery disease; CI = confidence interval; CT = computed tomography; ESUS = embolic stroke of undetermined origin; MRI = magnetic resonance imaging; SMD = standardized mean difference. * = most severely stenotic side; ** = side with stenosis <50%.

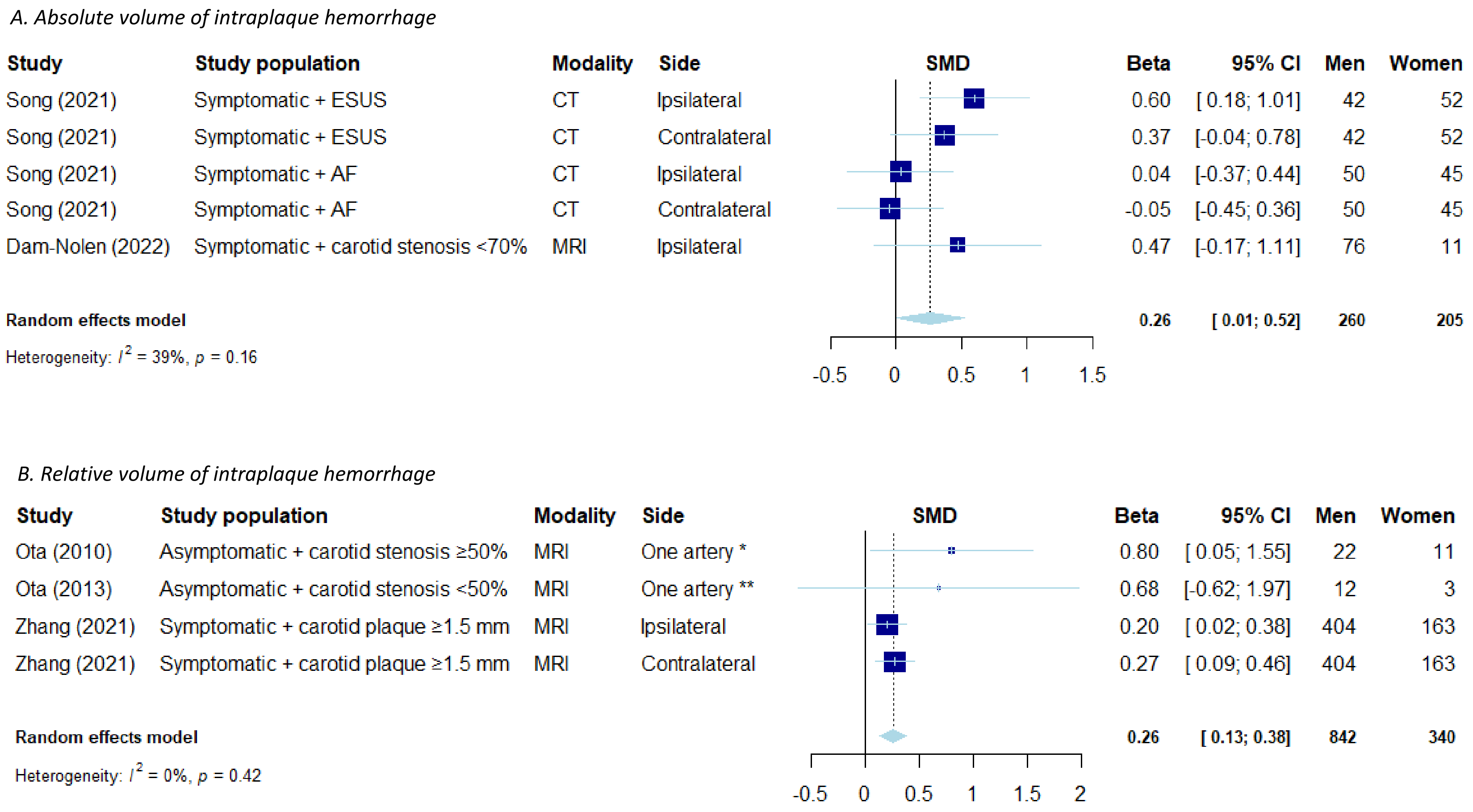

Figure S5. Meta-analyses on the association between sex and amount of intraplaque hemorrhage

The size of the box which represents the beta, is proportional to the weight of the study. The diamond is the result of the random-effect meta-analysis. Abbreviations: AF = atrial fibrillation; CI = confidence interval; CT = computed tomography; ESUS = embolic stroke of undetermined origin; MRI = magnetic resonance imaging; SMD = standardized mean difference.

* = most severely stenotic side; ** = side with stenosis <50%.

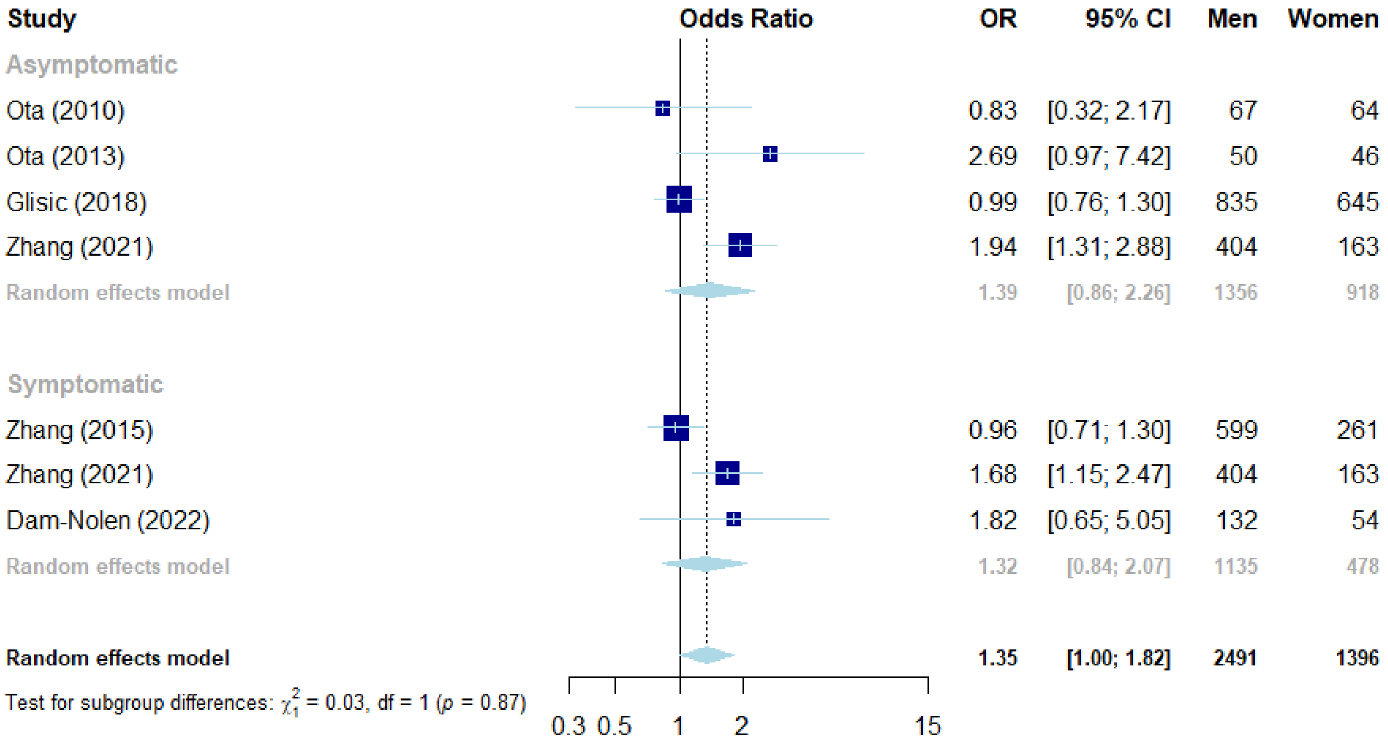

Figure S6. Stratified meta-analyses on the association between sex and calcifications

The size of the box which represents the odds ratio, is proportional to the weight of the study. The diamond is the result of the random-effect meta-analysis. Abbreviations: CI = confidence interval; OR = odds ratio.

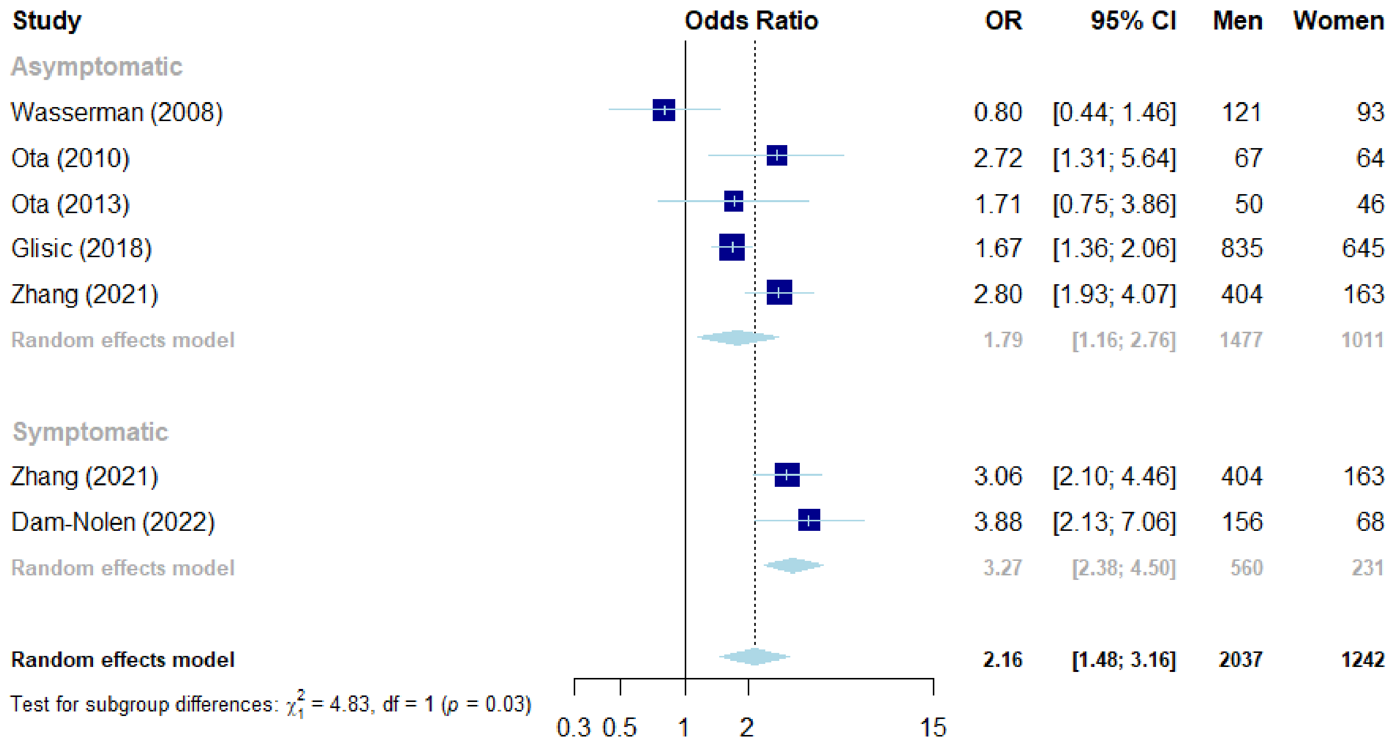

Figure S7. Stratified meta-analyses on the association between sex and lipid-rich necrotic core

The size of the box which represents the odds ratio, is proportional to the weight of the study. The diamond is the result of the random-effect meta-analysis. Abbreviations: CI = confidence interval; OR = odds ratio.

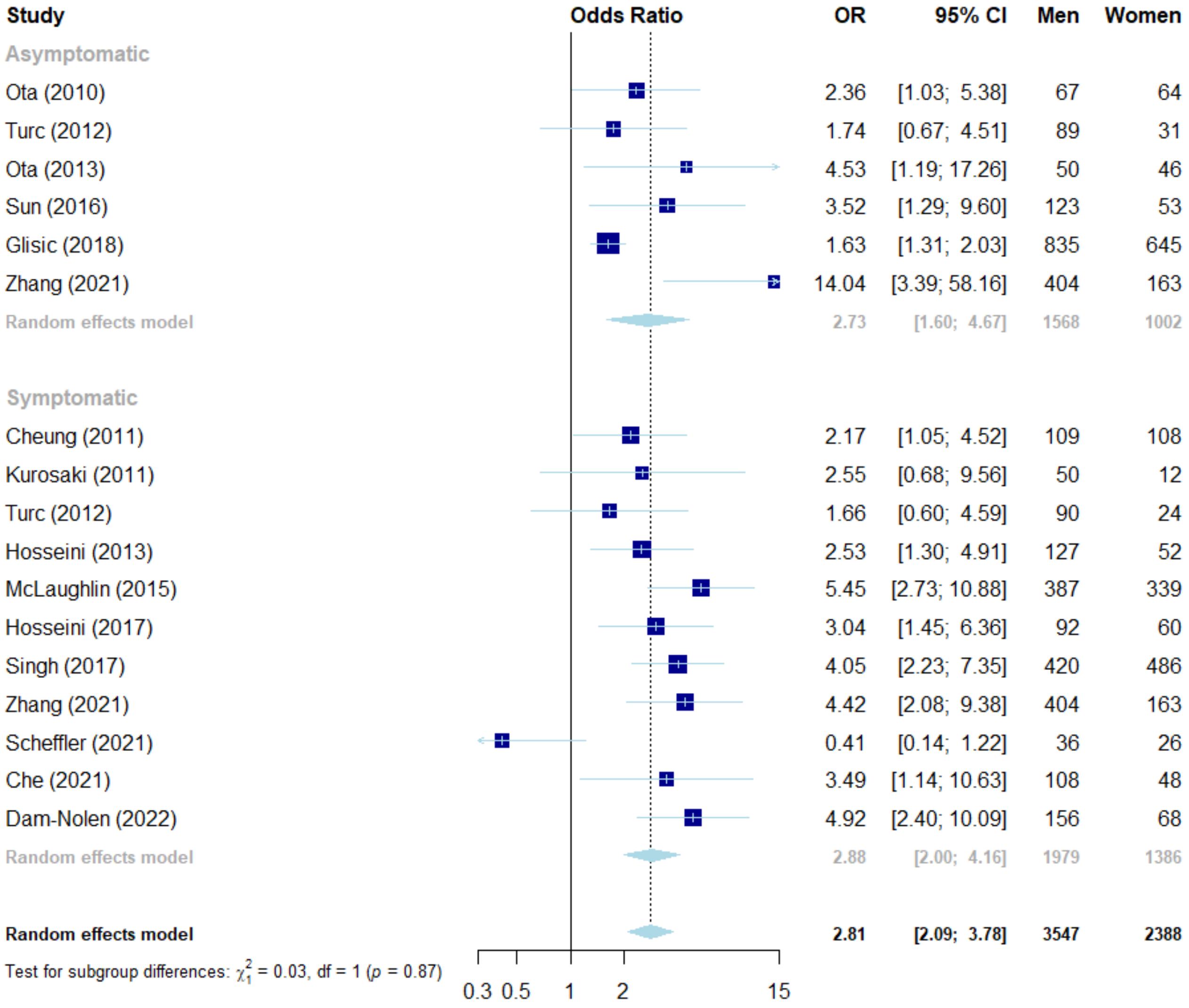

Figure S8. Stratified meta-analyses on the association between sex and intraplaque hemorrhage

The size of the box which represents the odds ratio, is proportional to the weight of the study. The diamond is the result of the random-effect meta-analysis. Abbreviations: CI = confidence interval; OR = odds ratio.

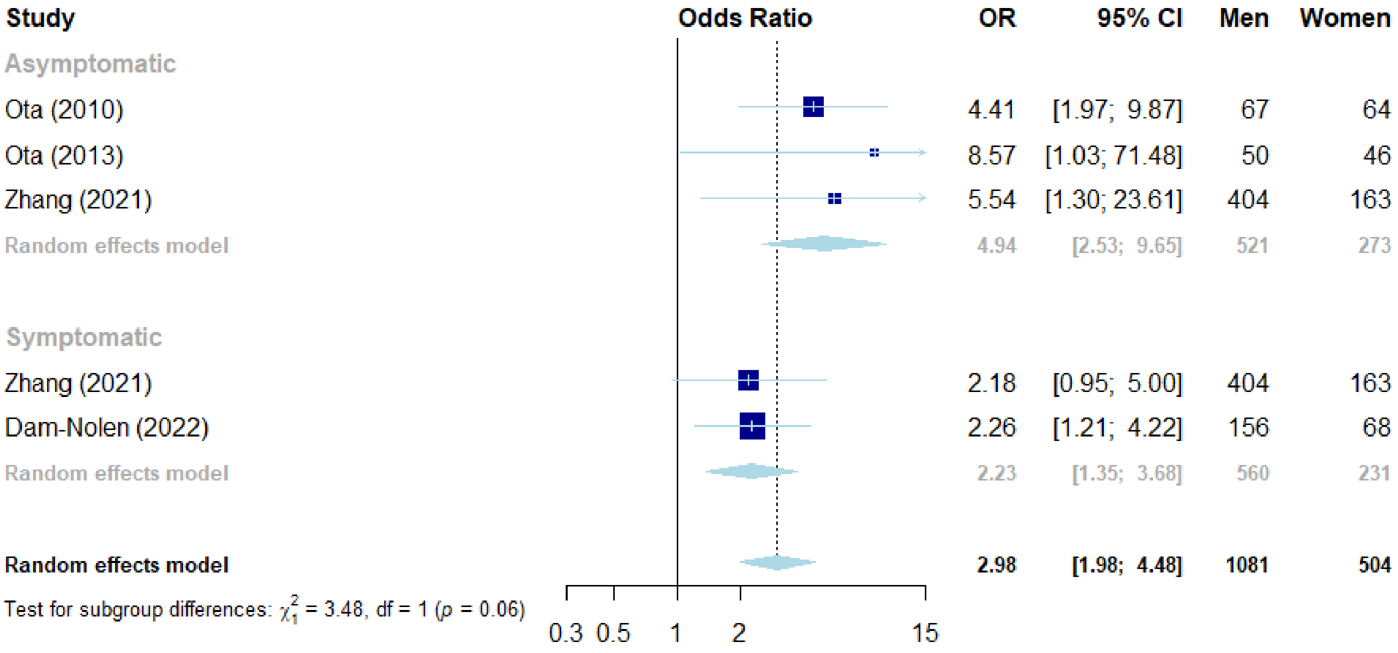

Figure S9. Stratified meta-analyses on the association between sex and thin-or-ruptured fibrous cap

The size of the box which represents the odds ratio, is proportional to the weight of the study. The diamond is the result of the random-effect meta-analysis. Abbreviations: CI = confidence interval; OR = odds ratio.
